# Hydride Transfer Mechanism of Enzymatic Sugar Nucleotide C2 Epimerization Probed with a Loose-Fit CDP-Glucose Substrate

**DOI:** 10.1021/acscatal.2c00257

**Published:** 2022-05-25

**Authors:** Christian Rapp, Bernd Nidetzky

**Affiliations:** † Institute of Biotechnology and Biochemical Engineering, Graz University of Technology, Petersgasse 10-12/1, 8010 Graz, Austria; ‡ Austrian Centre of Industrial Biotechnology (ACIB), Petersgasse 14, 8010 Graz, Austria

**Keywords:** protein dynamics and catalysis, sugar nucleotide epimerases, kinetic isotope effect, quantum mechanical tunneling, donor−acceptor distance (DAD), short-chain dehydrogenase/reductase (SDR)

## Abstract

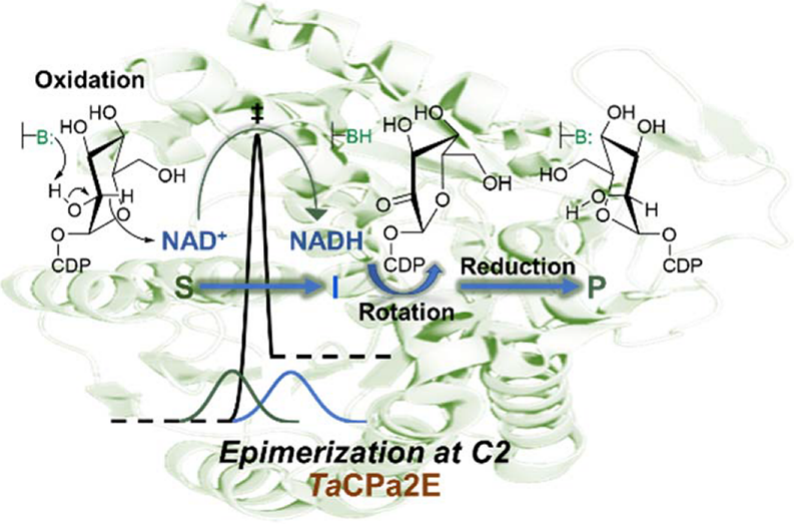

Transient oxidation–reduction through hydride transfer with tightly bound NAD coenzyme is used by a large class of sugar nucleotide epimerases to promote configurational inversion of carbon stereocenters in carbohydrate substrates. A requirement for the epimerases to coordinate hydride abstraction and re-addition with substrate rotation in the binding pocket poses a challenge for dynamical protein conformational selection linked to enzyme catalysis. Here, we studied the thermophilic C2 epimerase from *Thermodesulfatator atlanticus* (*Ta*CPa2E) in combination with a slow CDP-glucose substrate (*k*_cat_ ≈ 1.0 min^–1^; 60 °C) to explore the sensitivity of the enzymatic hydride transfer toward environmental fluctuations affected by temperature (20–80 °C). We determined noncompetitive primary kinetic isotope effects (KIE) due to ^2^H at the glucose C2 and showed that a normal KIE on the *k*_cat_ (^D^*k*_cat_) reflects isotope sensitivity of the hydrogen abstraction to enzyme-NAD^+^ in a rate-limiting transient oxidation. The ^D^*k*_cat_ peaked at 40 °C was 6.1 and decreased to 2.1 at low (20 °C) and 3.3 at high temperature (80 °C). The temperature profiles for *k*_cat_ with the ^1^H and ^2^H substrate showed a decrease in the rate below a dynamically important breakpoint (∼40 °C), suggesting an equilibrium shift to an impaired conformational landscape relevant for catalysis in the low-temperature region. Full Marcus-like model fits of the rate and KIE profiles provided evidence for a high-temperature reaction via low-frequency conformational sampling associated with a broad distribution of hydride donor–acceptor distances (long-distance population centered at 3.31 ± 0.02 Å), only poorly suitable for quantum mechanical tunneling. Collectively, dynamical characteristics of *Ta*CPa2E-catalyzed hydride transfer during transient oxidation of CDP-glucose reveal important analogies to mechanistically simpler enzymes such as alcohol dehydrogenase and dihydrofolate reductase. A loose-fit substrate (in *Ta*CPa2E) resembles structural variants of these enzymes by extensive dynamical sampling to balance conformational flexibility and catalytic efficiency.

## Introduction

Conformational selection enabled by protein flexibility is fundamental to enzyme catalysis.^1−5^ Directed changes in protein conformation (“coupled motions”) enable enzymes to coordinate the immediate catalytic event with other physical steps of the reaction, such as substrate binding and product release.^4−11^ In the chemical transformation on the enzyme, stochastic motions enable the dynamical population (“sampling”) of catalytically relevant ground-state conformers to have electrostatics and internuclear distances tuned for bond cleavage/formation.^12−19^ On this dynamic view, conformational selection connects directly to catalytic rate enhancement; and protein flexibility represents an evolutionary target for the optimization of enzyme efficiency.^20−22^ Enzymes of the alcohol dehydrogenase class (ADHs)^8,23−26^ as well as several other oxidoreductases (e.g., dihydrofolate reductase,^2,15,27−31^ thymidylate synthase,^32,33^ formate dehydrogenase,^34,35^ flavin-dependent ene-reductases,^36−39^ lipoxygenases^40,41^) have been instrumental to link conformational selection to catalysis. ADHs promote hydride transfer between the substrate and nicotinamide coenzyme.^5,24,25,42^ Protein flexibility enables ADHs to sample conformers that place the hydride donor in close proximity to the acceptor.^2,23,43−47^ Reaction occurs classically over the enthalpic barrier but also by quantum mechanical tunneling.^27,48−51^ Conformational selection of donor–acceptor distances (DADs) suitable for tunneling emphasizes dynamic control of the barrier width, in addition to a decrease in the barrier height, as an important element of the ADH catalysis.^43,45,48,52−58^ Extended ADH-type reactions that involve hydride transfer oxidation–reduction in multiple bond-breaking/forming steps pose a conundrum for the enzymes to achieve a well-tuned balance between protein flexibility and catalytic efficiency.^59−61^ Here, we explored dynamical features of catalytic hydride transfer in the context of sugar nucleotide epimerization.

**Scheme 1:**
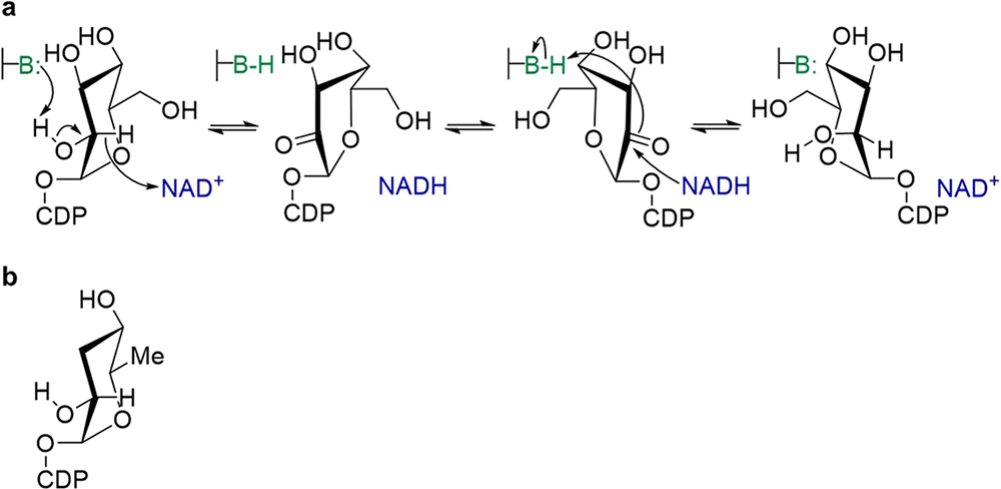
Epimerization of Sugar Nucleotide Substrates via Transient Oxidation–Reduction Epimerization shown for the conversion of CDP-glucose into CDP-mannose (a) catalyzed by *Ta*CPa2E (the C2 epimerase from *T. atlanticus*). CDP-paratose (b) is probably the native substrate of *Ta*CPa2E. It is shown for comparison with CDP-glucose.^62^

Transient oxidation–reduction via hydride transfer to and from the tightly bound NAD coenzyme is used by a large class of sugar nucleotide epimerases to invert carbon stereocenters in carbohydrate substrates (Scheme 1).^61−66^ The reaction starts with the oxidation of the alcohol group at the targeted stereocenter. Redelivery of the abstracted hydride from enzyme-NADH to the opposite face of the carbonyl in a suitably repositioned keto-intermediate gives the stereo-inverted product.^59,61^ The epimerases are unusual among enzymes in their requirement to be non-stereospecific.^59,65,67,68^ Their catalysis involves the stabilization of two stereoisomeric transition states for reversible cleavage of C–H bonds at carbon stereocenters. Abstraction and re-addition of the hydride are coordinated with rotation of the transient intermediate in the enzyme binding pocket.^59,65,69^ These main elements of epimerase catalysis present a significant challenge for enzyme conformational selection: protein flexibility necessary for the rotation must be aligned with precise positioning of the hydride donor and acceptor in tunneling-ready conformers. Notably, UDP-galactose 4-epimerase binds the 4-keto-pyranosyl moiety of the transient intermediate much more loosely than the corresponding UDP moiety (ΔΔ*G* = −5 kcal/mol).^61^ The considerations give rise to the suggestion that catalytic hydride transfer in the epimerase might involve a donor–acceptor distance sampling mode distinct from that of “simple” ADHs. A kinetic isotope effect (KIE) study was designed here to assess the mechanistic implication that the epimerase-catalyzed hydride transfer might be rather sensitive toward environmental fluctuations affected by the temperature. KIEs and their temperature dependence can serve as probes of protein motions that affect the C–H bond activation in enzymatic hydride transfer reactions.^13,27,33,34,70,71^

The thermophilic C2 epimerase from *Thermodesulfatator atlanticus* (*Ta*CPa2E)^72,73^ was investigated here in combination with a slow CDP-glucose (CDP-Glc) substrate (Scheme 1a). From its sequence, *Ta*CPa2E belongs to the group of CDP-paratose/CDP-tyvelose epimerases^62,66,74^ and is a member of the short-chain dehydrogenase/reductase (SDR) protein superfamily.^75−77^ The enzyme is a homodimer and each subunit contains tightly bound NAD^+^.^72^ The natural substrate of *Ta*CPa2E is probably CDP-paratose (Scheme 1b), which is 3,6-dideoxygenated compared with CDP-Glc. Reactivity of *Ta*CPa2E with CDP-paratose has not been determined, but the singly deoxygenated CDP-6-deoxy-glucose is ∼5-fold more active than CDP-Glc.^72^ The reason to select *Ta*CPa2E was the ability to specifically interrogate the enzymatic epimerization (CDP-glucose → CDP-mannose) over a broad temperature range (20–80 °C). Seminal research of ADHs^25,78^ and also dihydrofolate reductases^79−82^ has shown that a thermophilic enzyme can offer unique opportunities toward the aim of correlating protein flexibility to the nature of the chemical steps of catalysis. Moreover, the native flexibility of *Ta*CPa2E was unlikely to be optimized for the non-physiological CDP-glucose substrate (Scheme 1). We considered that the “loose fit” CDP-glucose might be instrumental to receive a mechanistically instructive temperature dependence of the KIE.

Evidence is presented that connects changes in the conformational landscape experienced by *Ta*CPa2E in response to the change in temperature with properties of the hydride transfer during transient substrate oxidation, which is shown to be rate-limiting overall. Interestingly, later steps of the catalytic cycle (i.e., keto-intermediate rotation and reduction from enzyme-NADH) do not affect the steady-state rate. Substrate activation for C–H bond cleavage requires partial deprotonation of the glucose 2-OH by an active-site base (Tyr164). Reduced protein flexibility in the low temperature range (≤40 °C) appears to restrict the efficiency of dynamical sampling, via coupled motion, for substrate activation. A model of temperature-dependent equilibration of differently active conformational substates in the conformational ensemble sampled by the enzyme–substrate complex is used to explain an unusual, and to our knowledge unique, kinetic characteristic of the *Ta*CPa2E: the observable KIEs are decreased progressively upon cooling down in the low-temperature region.

## Results

### C2 Epimerization of CDP-Glucose through Hydrogen Abstraction and Re-addition

**Figure 1:**
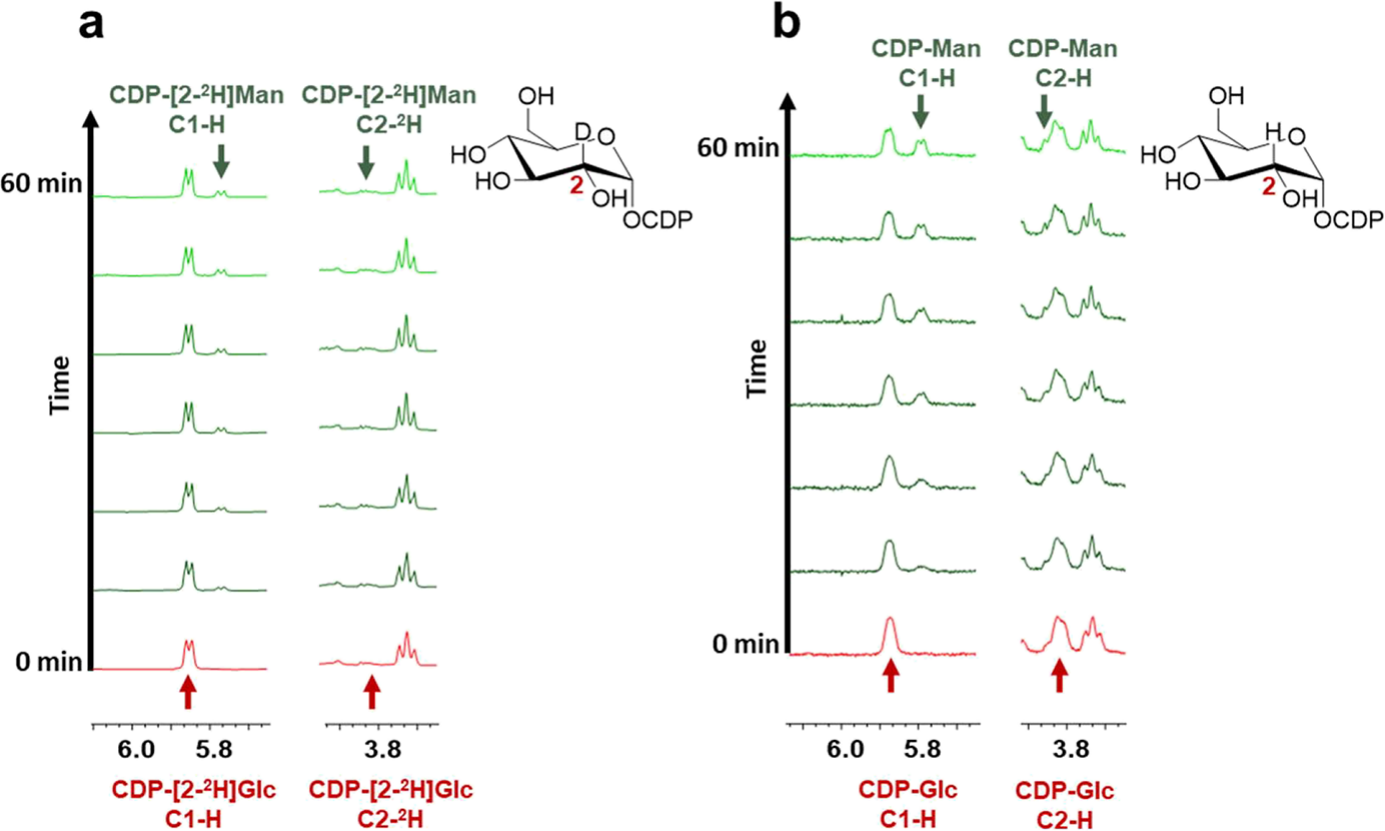
In situ ^1^H NMR measurements using *Ta*CPa2E and CDP-[2-^2^H]Glc (a) or CDP-Glc (b). Only selected spectra (recorded in 10 min intervals) are shown. Signals for C2 (3.80–3.86 ppm) and the anomeric region (5.75–5.90 ppm) are highlighted for both substrate (red) and product (green). The lack of signal in panel (a) at 3.82 ppm for CDP-[2-^2^H]Glc/Man stems from deuteride incorporation. For the full spectrum, see Figure S4. Reaction conditions: CDP-[2-^2^H]Glc (2.00 mM) and 50.8 μM *Ta*CPa2E (2.0 mg/mL); CDP-Glc (4.00 mM) and 15.2 μM *Ta*CPa2E (0.6 mg/mL); 60 °C, 50 mM potassium phosphate buffer (p^2^H = 7.5).

To verify the proposed reaction mechanism of *Ta*CPa2E (Scheme 1), CDP-[2-^2^H]glucose (CDP-[2-^2^H]Glc) was synthesized and underwent enzymatic conversion into CDP-mannose (CDP-Man) analyzed by in situ proton NMR in ^2^H_2_O solvent (p^2^H = 7.5). Time-resolved spectra from the reaction (Figure 1a) showed that the deuterium label at the C2 of the CDP-glucose substrate was retained completely in the CDP-mannose product. Reference reaction with CDP-[^1^H]glucose showed that deuterium was not incorporated from the solvent (Figure 1b). These results confirmed CDP-glucose C2 epimerization via hydrogen abstraction from, and re-addition to, the C2 of the d-hexopyranosyl moiety.

The enzymatic cycle of oxidation–reduction requires catalytic facilitation from a general acid–base, as indicated in Scheme 1. Based on known structure–function relationships of SDR-type epimerases,^65,75,83^ Tyr164 was the clear candidate residue of *Ta*CPa2E to fulfill that role. The Y164F variant was generated to replace the tyrosine with a residue minimally disruptive structurally but was incompetent in the proposed catalysis. The purified variant contained tightly bound NAD^+^. In activity assays for a range of protein concentrations (25.4–127 μM) at 60 °C and pH 7.5, the Y164F variant was inactive to epimerize CDP-glucose above the detection limit (≥10^4^-fold decrease in a specific rate compared to wild-type *Ta*CPa2E). Reactions at higher pH (up to 9.5) did not elicit epimerase activity, which was considered possible in the case that the Y164F variant enabled specific base catalysis from H_2_O/OH^–^ similarly to how an analogous Tyr → Phe variant of UDP-glucuronic acid 4-epimerase did.^64^ Collectively, the evidence suggested a classical SDR mechanism (Scheme 1) of C2 epimerization of CDP-glucose by *Ta*CPa2E.

### Transient Oxidation of the Substrate Is Rate-Limiting for Overall Epimerization of CDP-Glucose

The noncompetitive KIE on the substrate-saturated rate (*k*_cat_) at 60 °C and pH 7.5 (Figure S1) was determined as 4.3 ± 0.3 (*N* = 4). The large value of ^D^*k*_cat_ implied a substantial contribution from the C–H bond breaking/forming steps of catalysis to the overall rate limitation of enzymatic epimerization. Full Michaelis–Menten kinetics were therefore recorded to also obtain the KIE on the substrate-limited rate (*k*_cat_/*K*_M_). Data were acquired at 60 °C and additionally at the upper and lower limit of the temperature range (20–80 °C) considered for study of the temperature effect on the enzymatic rate (see later). The results are summarized in Table 1. At each temperature, the KIE on the *k*_cat_/*K*_M_ was identical within the limits of error of the KIE on the *k*_cat_. This evidence strongly supports the idea of rate limitation by the isotope-sensitive steps of catalysis (for the general case, see ref (84)), independent of the temperature varied between 20 and 80 °C.

**Table 1:** Kinetic Parameters and their Corresponding Primary KIEs for the Epimerization of CDP-Glc at Distinct Temperatures^a^

*T* (°C)	substrate	*k*_cat_ (min^–1^)	*K*_M_ (mM)	^D^*k*_cat_	^D^*k*_cat_/*K*_M_
20	CDP-Glc	(1.20 ± 0.06) × 10^–2^	0.24 ± 0.05	2.20 ± 0.75	2.06 ± 0.67
	CDP-[2-^2^H]Glc	(0.62 ± 0.02) × 10^–2^	0.25 ± 0.03		
60	CDP-Glc	0.88 ± 0.03	0.30 ± 0.04	4.30 ± 0.30	3.80 ± 0.40
	CDP-[2-^2^H]Glc	0.21 ± 0.01	0.26 ± 0.03		
80	CDP-Glc	1.31 ± 0.04	0.16 ± 0.03	3.34 ± 0.09	3.19 ± 0.24
	CDP-[2-^2^H]Glc	0.40 ± 0.02	0.15 ± 0.04		

a The *k*_cat_ and *K*_M_ values were determined by a non-linear fit to the specific rates (*V*/[E], min^–1^) dependent on the substrate concentration. Data for the *protio* and *deuterio* substrate were fitted separately. The KIEs on *k*_cat_ (^D^*k*_cat_) and on *k*_cat_/*K*_M_ (^D^*k*_cat_/*K*_M_) were obtained by a global nonlinear fit to the data for both isotopologue substrates. The procedures used are described under Materials and Methods.

**Figure 2:**
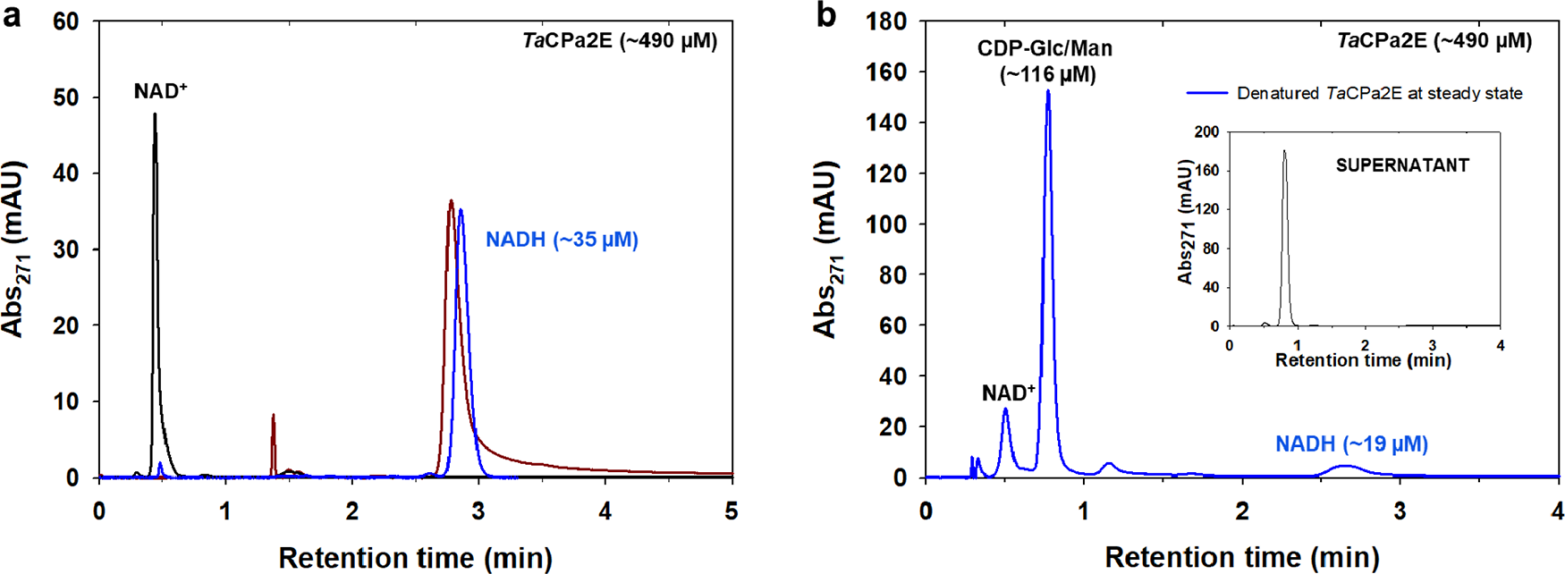
Determination of the steady-state enzyme-NADH in the epimerization of CDP-Glc by *Ta*CPa2E. (a) Overlay of HPLC chromatograms displaying denatured *Ta*CPa2E prior to the reaction (blue), NAD^+^ standard (black), and NADH standard (brown). (b) HPLC chromatogram of denatured *Ta*CPa2E from the reaction at the steady state, showing the enzyme-bound NADH and substrate/product mixture (blue). The NAD^+^ coenzyme is partially released. The inset shows the reaction mixture after 15 min when quenched with methanol and the enzyme removed. The injection volume was half compared to (a). For NADH quantification and method validation, see Figure S5 and Materials and Methods.

There are two isotope-sensitive steps in the mechanism, one in each half-reaction (Scheme 1). At the steady state of the reaction, the relative rates of transient oxidation and reduction define the portion of total enzyme present in the reduced (NADH) form. Using a rapid-quench assay previously developed for UDP-glucuronic acid 4-epimerase^64^ and here adapted to *Ta*CPa2E (Figure 2), we determined the portion of enzyme-NADH as 2.22% (± 0.5%; *N* = 4), which was not increased compared to the NADH content of the resting enzyme in the absence of the substrate (4.15 ± 1.0%; *N* = 4).

Taken in combination, therefore, the high ^D^*k*_cat_ (identical with ^D^*k*_cat_/*K*_M_) and the low steady-state portion of enzyme-NADH indicated that the observed KIE arose from C–H bond cleavage in CDP-glucose during rate-limiting transient oxidation of the substrate. The measurement of enzyme-NADH was from the initial-rate phase when the CDP-glucose conversion was just 7–8% of the reaction equilibrium. The *K*_eq_ (= [CDP-[2-^2^H]mannose]^eq^/[CDP-[2-^2^H]glucose]^eq^) was 0.67 under the conditions used (60 °C, pH 7.5, Figure S6). The equilibrium position was independent of the 2-^2^H isotopic substitution of the CDP-glucose substrate (^D^*K*_eq_ ≈ 1.0). The ratio of CDP-mannose/CDP-glucose associated with the enzyme from rapid-quench processing was 0.163 (± 0.008; *N* = 4). The corresponding product/substrate ratio in bulk solution was 0.100 (± 0.004; *N* = 4). Evidence that the enzyme-bound product/substrate ratio was considerably lower than the external equilibrium determined by the *K*_eq_ while it was similar to the product/substrate ratio in solution was good support in favor of the suggestion that the kinetic mechanism of *Ta*CPa2E-catalyzed epimerization of CDP-glucose involved rate-limiting oxidation of the substrate.

### Temperature Dependence of *k*_cat_

**Figure 3:**
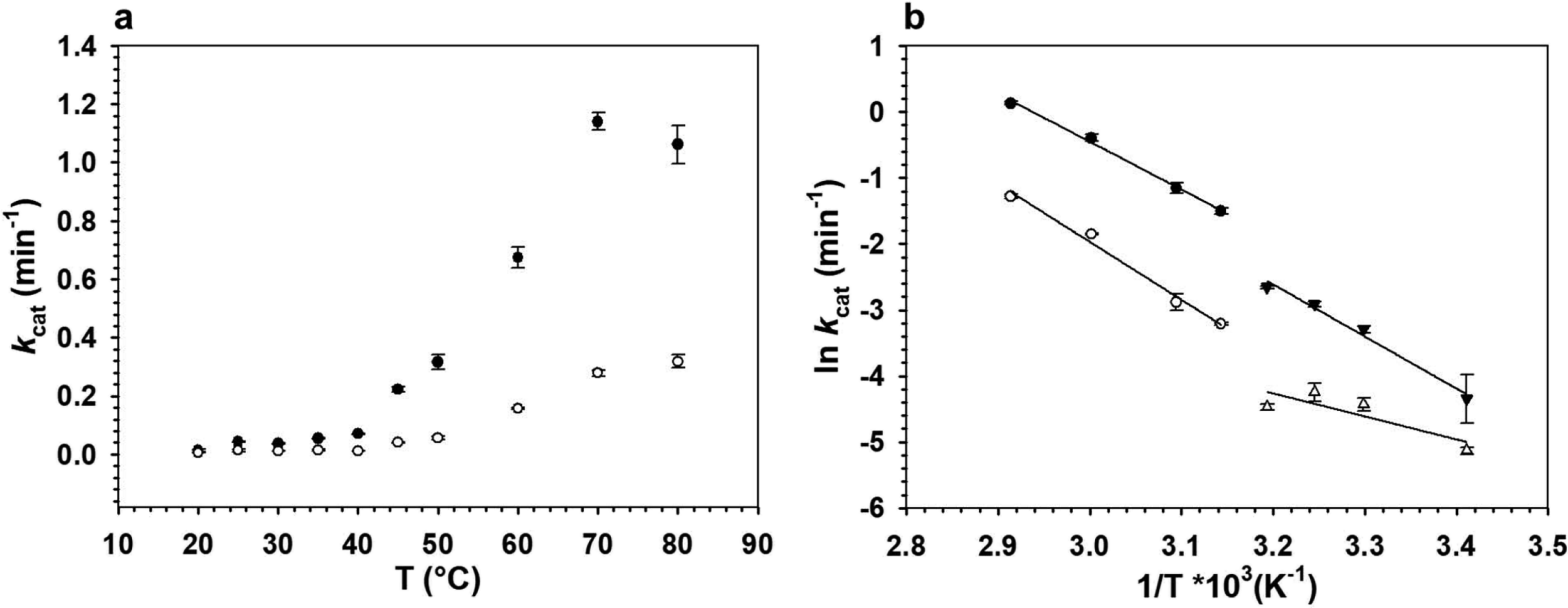
Temperature dependence of *k*_cat_ ((a), temperature profile; (b) Arrhenius plots) for the reaction of *Ta*CPa2E with CDP-Glc and CDP-[2-^2^H]Glc. Epimerizations were analyzed in a temperature range of 20–80 °C using *Ta*CPa2E (1.0 mg/mL; 25.4 μM) and 4.00 mM CDP-Glc (black dots, black triangles) or CDP-[2-^2^H]Glc (white circles, white triangles). Due to a low coefficient of determination, results for 25 and 80 °C were omitted in (b). Symbols show the experimental data, and error bars show the associated S.D. (*N* = 4). The lines in panel (b) are straight-line fits with the Arrhenius model (eq 1).

Temperature profiles of *k*_cat_ for the reaction with 2-[^1^H]- and 2-[^2^H]-CDP-glucose in the range 20–80 °C (pH 7.5) are displayed in Figure 3. With either substrate, the profile showed a prominent break (abrupt increase in the *k*_cat_ by ∼3-fold) in going from 40 to 45 °C. Above and below the break, the profiles featured “Arrhenius-like” (eq 1) behavior and were fitted accordingly (Figure 3), with parameter estimates summarized in Table 2. A possible deviation from linearity is however noted for the Arrhenius plot of the *k*_cat_ for the reaction with 2-[^2^H]-CDP-glucose in the low temperature range (≤40 °C). Clear discrimination between linear and curved dependence was not possible in this range.
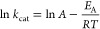
1where *k*_cat_ is the rate constant (min^–1^), *T* is temperature [K], *R* is the gas constant [kJ mol^–1^ K^–1^], *A* is an Arrhenius prefactor, and *E*_A_ is the activation energy [kJ mol^–1^].

**Table 2:** Activation Energies *E*_A_ and Arrhenius Prefactors *A* Obtained for CDP-Glc and CDP-[2-^2^H]Glc for the Temperature Range of 20–40 °C and 45–70 °C^a^

	20–40 °C		45–70 °C	
substrate	*E*_A_ (kJ mol^–1^)	*A* (min^–1^)	*E*_A_ (kJ mol^–1^)	*A* (min^–1^)
CDP-Glc	+66.7 ± 9.4	(6.9 ± 1.2) × 10^9^	+60.2 ± 3.3	(1.7 ± 0.3) × 10^9^
CDP-[2-^2^H]Glc	+29 ± 4.5	(9.9 ± 2.6) × 10^2^	+72.9 ± 6.7	(3.8 ± 0.7) × 10^10^
Δ*E*_A_	–36.7 ± 7.6		+12.7 ± 2.6	
*A*_H_/*A*_D_	(7.0 ± 1.1) × 10^6^		0.05 ± 0.007	

a *E*_A_ and *A* (*y* intercept) were obtained from the slope of the linearized Arrhenius equation (see eq 1). The activation energy difference Δ*E*_A_ (= *E*_A,D_ – *E*_A,H_) and Arrhenius prefactor ratios *A*_H_/*A*_D_ were calculated accordingly. Subscript H and D indicate parameters for the reaction with the *protio* and *deuterio* substrate, respectively.

**Figure 4:**
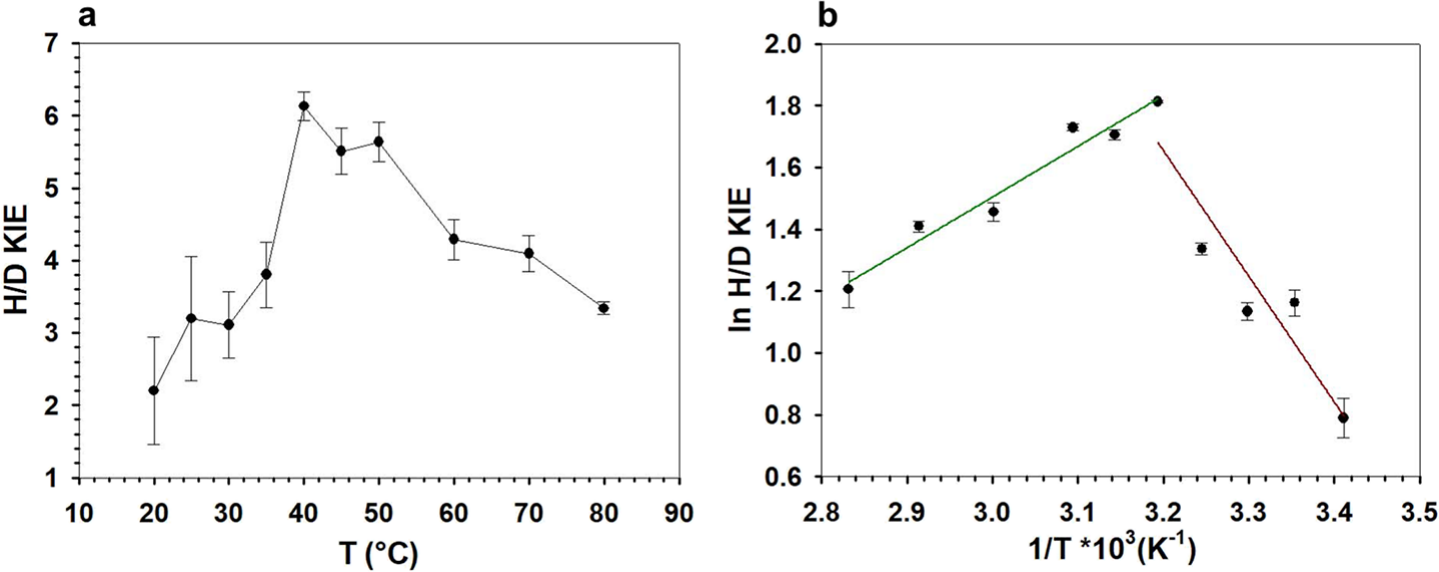
Temperature dependence of the KIE on *k*_cat_ for *Ta*CPa2E. (a) H/D KIE dependent on temperature *T* (°C) and (b) ln H/D KIE dependent on reciprocal *T* (1/K). In panel (b), two temperature regimes are highlighted (green: 40–80 °C; brown: 20–40 °C). The straight lines are linear fits of the data. Symbols show the experimental data, and error bars show the associated S.D. (*N* = 4).

From the results in Figure 3, we also obtained a temperature profile of the KIE, as shown in Figure 4. The ^D^*k*_cat_ peaked at 40 °C, with its value of 6.1 approaching the semiclassical limit (∼6.9) of the primary deuterium KIE.^33,85^ The result suggested that the chemical step of C–H bond cleavage in the substrate had become unmasked strongly in the measured rate parameter under the conditions used. Figure 4 shows that the ^D^*k*_cat_ declined continuously in the temperature ranges above and below the 40 °C peak. We discuss later that the ^D^*k*_cat_ decline at low temperature was not consistent with the anticipated effect of environmental cooling on the intrinsic hydride transfer. To clarify whether the decrease in ^D^*k*_cat_ could have arisen from a change in the location of the rate-limiting step, we determined the portion of enzyme-NADH at the steady state during the enzymatic reaction at 20 °C. Enzyme-NADH was still present in low amounts (∼3%) and not elevated as compared to the as-isolated *Ta*CPa2E in the absence of the substrate. The result is consistent with the KIE data in Table 1, showing that for the reaction at 20 °C, ^D^*k*_cat_ was identical to ^D^*k*_cat_/*K*_M_.

### Solvent Isotope Effects

Considering the requirement of proton transfer in the enzymatic oxidation of CDP-Glc (Scheme 1a), we analyzed the effect of solvent deuteration (^2^H_2_O) on the *k*_cat_ for the reactions with CDP-Glc and CDP-[2-^2^H]Glc. The results are summarized in Table 3. Control reactions in ^1^H_2_O in the presence of 9% v/v glycerol showed no effect on the *k*_cat_ (±3%). The result rules out the assumption that the enhanced solvent microviscosity of ^2^H_2_O compared to ^1^H_2_O could have influenced the measured rates.^86^ With both substrates, the *k*_cat_ at 60 °C was independent of the pL (L = ^1^H or ^2^H) in the range 7.0–8.0. The solvent isotope effect (SKIE) on *k*_cat_ (^D_2_O^*k*_cat_) was therefore also independent of the pL in the range analyzed. Using the *protio* substrate, the ^D_2_O^*k*_cat_ was not different from unity within the limits of the experimental error. Using the *deuterio* substrate, however, the ^D_2_O^*k*_cat_ was inverse (0.57 ± 0.11; pL 7.5). The KIE from deuteration of the CDP-Glc was therefore solvent-dependent. It was 4.30 (± 0.30) in ^1^H_2_O as already mentioned, and it was 2.34 (± 0.27) in ^2^H_2_O. The lowering of the ^D^*k*_cat_ in ^2^H_2_O could arise if there was a step in the enzymatic mechanism, different from the catalytic step of hydride abstraction to NAD^+^, that was sensitive to solvent deuteration and so became partly rate-limiting in ^2^H_2_O. To explore this possibility, we measured the reduced portion of total enzyme at the steady state during the reaction in ^2^H_2_O at 60 °C. The NADH content in the enzyme sample from the reaction was 1.90% (± 0.60%; *N* = 4). This was almost identical to the NADH content of the resting enzyme in ^2^H_2_O (2.33 ± 0.88%; *N* = 4). Moreover, the results in ^2^H_2_O were very similar to the ones in ^1^H_2_O. Therefore, the evidence implies that the ^D_2_O^*k*_cat_ in the reaction with CDP-[2-^2^H]Glc was due to a solvent effect on the transient oxidation catalyzed by the enzyme.

**Table 3:** Solvent Kinetic Isotope Effects on *k*_cat_ for Reactions of *Ta*CPa2E with CDP-Glc and CDP-[2-^2^H]Glc at Varied Temperatures and pL Values

substrate	^D_2_O^*k*_cat_ (pL 7.5) 80 °C	^D_2_O^*k*_cat_ (pL 7.0) 60 °C	^D_2_O^*k*_cat_ (pL 7.5) 60 °C	^D_2_O^*k*_cat_ (pL 8.0) 60 °C	^D_2_O^*k*_cat_ (pL 7.5) 20 °C
CDP-Glc	n.d.^a^	0.97 ± 0.12	1.03 ± 0.13	0.99 ± 0.11	0.49 ± 0.07^b^
CDP-[2-^2^H]Glc	0.84 ± 0.03	0.57 ± 0.11	0.57 ± 0.10	0.59 ± 0.10	0.51 ± 0.10

a n.d., not determined.

b From a full Michaelis–Menten analysis performed in these conditions (Figure S3a,b), the ^D_2_O^*k*_cat_/*K*_M_ was determined as 0.65 (± 0.31).

The SKIE on the *k*_cat_ was additionally determined at low (20 °C) and high temperature (80 °C) where the ^D^*k*_cat_ decreases strongly, as shown before in Figure 4a. Using CDP-Glc, the ^D_2_O^*k*_cat_ was inverse (0.49 ± 0.07) at 20 °C, thus substantially different from the ^D_2_O^*k*_cat_ at 60 °C. Using CDP-[2-^2^H]Glc, the ^D_2_O^*k*_cat_ at 20 °C (0.51 ± 0.10) was similar to the ^D_2_O^*k*_cat_ at 60 °C. In contrast to 60 °C, therefore, the ^D^*k*_cat_ at 20 °C was not dependent on the solvent used. Using CDP-[2-^2^H]Glc at 80 °C, the ^D_2_O^*k*_cat_ (= 0.84 ± 0.03) was less strongly inverse than at 60 °C.

## Discussion

### Kinetic Mechanism of *Ta*CPa2E

**Scheme 2:**
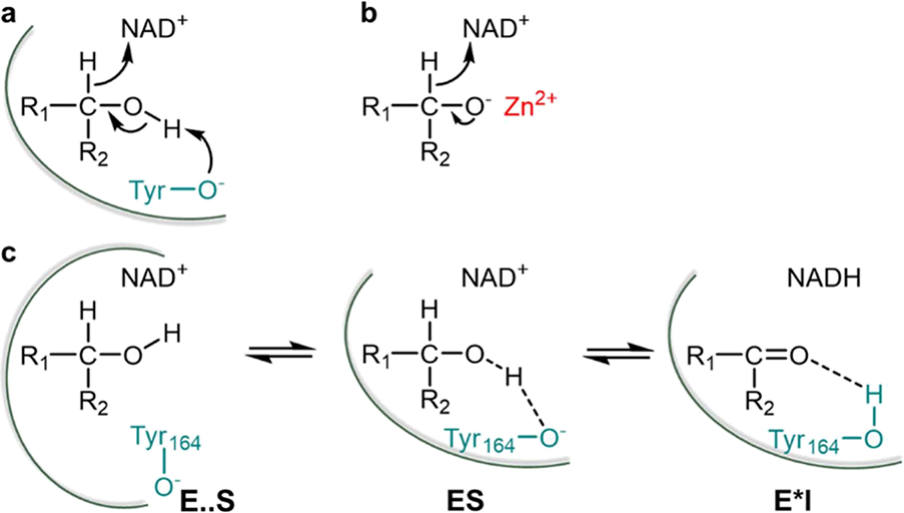
Enzyme Strategies to Facilitate Hydride Transfer Oxidation by Deprotonation of the Alcohol Substrate (a) General base catalysis, concerted reaction; (b) electrostatic stabilization by the metal ion, stepwise reaction; (c) two-step catalytic mechanism of substrate oxidation by *Ta*CPa2E, with an initial enzyme–substrate complex (E..S) undergoing coupled motions to populate the reactive enzyme–substrate conformer (ES) that converts chemically to the keto-intermediate complex E*I. A strong (low barrier-like) hydrogen bond may be characteristic of the ES complex, distinct from the “normal” hydrogen bond present in E*I.

Evidence was shown that transient oxidation of the substrate is rate-limiting on *k*_cat_ for the overall epimerization of CDP-glucose into CDP-mannose (Scheme 1a) over the entire temperature range studied. The chemical step of transient oxidation presumably involves coordinated (i.e., concerted but likely asynchronous^77,87−90^) abstraction of the hydride from the C2 and the proton of the 2-OH of the reactive alcohol group, as shown in Scheme 2. A reaction coordinate featuring overlapped timing of proton and hydride transfer is characteristic of metal-independent enzyme catalysis to alcohol oxidation.^91−93^ It differs from the stepwise catalysis of Zn^2+^-dependent ADHs.^94^ In these ADHs, lowering of the p*K*_a_ in the Zn^2+^-bound alcohol strongly activates the substrate for C–H bond cleavage already in the ground-state complex.^94^ Given this major difference in catalysis of the chemical step, the KIE analysis for *Ta*CPa2E was of interest to characterize the metal-independent mechanism of enzymatic alcohol oxidation. Plausible kinetic scenario for the epimerase involves two steps, whereby a relatively slow physical step with a time constant similar to that of catalysis precedes the chemical step of hydride abstraction to NAD^+^ (Scheme 2).^64,95−97^ The physical step likely involves coupled motions of the substrate-bound *Ta*CPa2E toward enzyme conformers competent to undergo catalytic conversion. An essential task of this physical step in catalysis would be the precise structural alignment of the substrate’s C2-OH and C2-H with the enzyme’s general base (the ionized side chain of Tyr164) and the C4 of NAD^+^, respectively, to promote the concerted reaction (Scheme 2c). The observable KIE on *k*_cat_ is analyzed with eq 2,
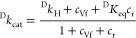
2where ^D^*k*_H_ is the isotope effect on the isotope-sensitive step, *c*_Vf_ is a constant that compares the rate constant for the isotope-sensitive step with rate constants for all other unimolecular forward steps, *c*_r_ is a so-called “commitment” for the reverse direction of the reaction, and ^D^*K*_eq_ is the equilibrium isotope effect on the step analyzed. The ^D^*K*_eq_ of hydride transfer oxidation of alcohols by NAD^+^ is in the range 1.1–1.2.^98−100^ Note that ^D^*k*_H_ is the intrinsic KIE of the chemical step partly masked by “internal” commitments, like the proposed precatalytic physical step in Scheme 2c.

We assume in the discussion to follow that the substantially inflated ^D^*k*_cat_ of 6.1 at 40 °C implies the intrinsic KIE to have largely been revealed under these conditions. The ^D^*k*_cat_ in the temperature range 40–80 °C was therefore considered to show the KIE on the chemical step intrinsically. The evolution of the KIE in the low-temperature regime was unusual and required clarification.

**Scheme 3:**
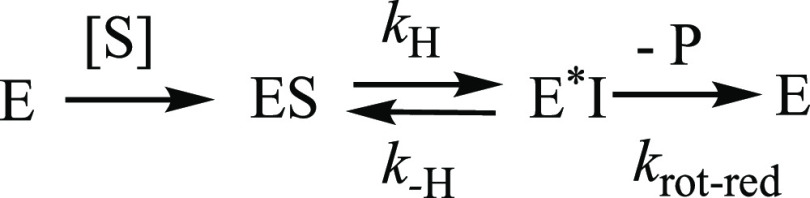
Proposed Minimal Kinetic Mechanism of *Ta*CPa2E Used to Analyze the Enzymatic Rates of CDP-Glc Consumption and Their Associated KIEs E and E* are enzyme-NAD^+^ and enzyme-NADH, respectively. I stands for the 2-keto intermediate and P for CDP-Man.

A minimal kinetic scheme consistent with the experimental evidence is Scheme 3, with the important addition that the net rate of conversion of ES into E*I is rate-determining. It follows that *c*_r_ = *k*_–H_/*k*_rot-red_ and *c*_Vf_ = *k*_H_/*k*_rot-red_, where *k*_–H_ is the rate constant for the reverse isotope-sensitive step and *k*_rot-red_ is a net rate constant for all further unimolecular steps, including rotation, reduction, and product release. Since ^D^*k*_cat_ is identical with ^D^*k*_cat_/*K*_M_ and no enzyme-NADH accumulates, the *c*_Vf_ must be small, with *k*_H_ ≪ *k*_rot-red_ as discussed. To account for the observed decrease in ^D^*k*_cat_ below 40 °C, the *c*_r_ would have to increase at low temperature, requiring that the *E*_A_ on *k*_rot-red_ be larger considerably than the *E*_A_ on *k*_–H_ According to Scheme 3, the *k*_cat_ depends on *k*_rot-red_ and *k*_–H_ as shown in eq 3a. With the additional assumption of *k*_H_ ≪ *k*_rot-red_, one gets the simpler eq 3b.
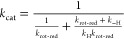
3a
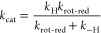
3b

The temperature dependence of the *k*_cat_ can be expressed by the combined Arrhenius equations of the rate constants, as shown in eq 4.
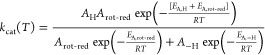
4

If as discussed above the steps included in *k*_rot-red_ involved a relatively high *E*_A_, *k*_cat_ would correspond to ∼*k*_H_ at high temperature and to ∼*k*_H_*k*_rot-red_/*k*_–H_ at low temperature. The apparent *E*_A_ associated with the *k*_cat_ at low temperature would thus be [*E*_A_(*k*_–H_) – *E*_A_(*k*_rot-red_) – *E*_A_(*k*_H_)]. Due to the requirement of *c*_r_ that *E*_A_(*k*_rot-red_) > *E*_A_(*k*_–H_), the difference term would yield a negative overall activation energy, which is unreasonable. We thus exclude the possibility that temperature dependence of *c*_r_ accounts for the decrease in ^D^*k*_cat_ at low temperature.

Next, we consider that the overall catalysis of hydride abstraction by NAD^+^ comprises two steps as indicated in Scheme 2c. The *k*_H_ would then be expressed by eq 5
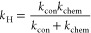
5where *k*_con_ is a precatalytic step (E..S → ES) and *k*_chem_ is the chemical step of catalysis (ES → E*I). Since *k*_con_ is not isotope-sensitive, it must be the same for the *protio* and the *deuterio* substrate. A decrease in the observable KIE on the rate caused by the “kinetic complexity” of *k*_H_ must arise from *E*_A_(*k*_con_) larger than *E*_A_(*k*_chem_). At low temperature, therefore, the *k*_H_ would gradually become ∼*k*_con_. The Arrhenius plot for *k*_cat_ (≈*k*_H_) would be curved downward toward a limiting *E*_A_(*k*_con_). The experimental results for *k*_cat_ with both CDP-Glc and CDP-[2-^2^H]Glc (Figure 3b) are not consistent with the predicted behavior. Overall, therefore, these considerations emphasize the requirement of a different mechanistic model to explain the low-temperature dependence of *k*_cat_ and ^D^*k*_cat_. A model based on conformational selection by the enzyme is proposed.

### Evidence from Solvent Isotope Effects

Interpretation of the SKIE data is based on the notion from the discussion above that the hydride transfer from substrate to enzyme-NAD^+^ is rate-limiting for the *k*_cat_ in the full temperature range from 20 to 80 °C. Evidence that the ^D_2_O^*k*_cat_ at 20 °C was identical with the *protio* and *deuterio* substrate thus suggests that both isotope effects (^D_2_O^*k*_cat_, ^D^*k*_cat_) arose from a single rate-limiting step. The implication that the ^D_2_O^*k*_cat_ is due to a solvent sensitivity of the immediate catalytic step renders the inverse nature of the SKIE of interest. The observable ^D_2_O^*k*_cat_ can be expressed from the deuterium fractionation factors Φ (i.e., the exchange equilibrium constant between ^1^H_2_O and ^2^H_2_O solvent) of the ground-state complex ES (Φ_GS_) and the relevant enzyme complex involved in the reaction barrier crossing, typically the transition state TS (Φ_TS_): ^D_2_O^*k*_cat_ = Φ_GS_/Φ_TS_. For the ^D_2_O^*k*_cat_ to become inverse, the somewhat unusual situation of Φ_GS_ < Φ_TS_ must apply. Groups responsible for inverse SKIEs in enzymatic reactions are cysteine (Φ ≈ 0.55^101^) and metal-bound water,^102^ but neither plays a role in the epimerase. An inverse SKIE of 0.6 ± 0.1 on *k*_cat_/*K*_M_ was found in the Claisen-like condensation of acetyl-coenzyme A and glyoxylate by malate synthase using the deuterated substrate.^103^ No SKIE was observed with the unlabeled substrate. The authors suggested that metal-bound water or global solvent reorganization enabling a conformational change upon substrate binding might explain these observations. Here, with *Ta*CPa2E, besides a pre-catalytic conformational equilibrium dependent on solvent, a strong (effectively low-barrier)^104^ hydrogen bond developed at the ground-state complex between the ionized Tyr164 and the 2-OH of the substrate (Scheme 2c, complex ES) could explain a Φ_GS_ considerably smaller than unity. The Φ of a negatively charged molecular group of the general form (RO–H–OR) is between 0.27 and 0.47 in a nonaqueous environment (CH_3_CN solvent).^101^ The Φ of a regular hydrogen bond, arguably present at the 2-keto intermediate state (Scheme 2c, complex E*I), is ∼1.0. The requirement of the Φ_TS_ to be the intermediate between the Φ_GS_ and the Φ_IS_ would imply a proton transfer that is advanced considerably and involves the hydride transfer lagging behind at the transition state of the reaction. The catalytic scenario suggested for the epimerase shows a striking analogy to Zn^2+^-ADH catalyzing oxidation of ethanol by NAD^+^: Φ_GS_ = 0.37 (low-barrier hydrogen bond); Φ_TS_ = 0.73; Φ_PS_ = 1 (aldehyde product state).^105,106^

The change in the ^D_2_O^*k*_cat_ with temperature, different for the *protio* and *deuterio* substrate, is difficult to interpret with confidence. However, a partially rate-limiting deprotonation of the substrate associated with the hydride transfer appears to be ruled out for the full temperature range examined. Sensitivity to solvent deuteration of the reaction barrier crossing by quantum mechanical tunneling, dependent on the temperature and substrate isotope, could be an interesting topic for further mechanistic study of the epimerase.

### Switch to an Impaired Conformational Landscape at Low Temperature

Both *k*_cat_ and ^D^*k*_cat_ of the *Ta*CPa2E reaction with CDP-glucose showed unusual dependence on the temperature, with abrupt breaks seen at ∼40 °C. Enzyme kinetic behavior at and below the temperature break arose from processes completely reversible upon raising the temperature back to above 40 °C. A plausible explanation for it was a change in the conformational landscape experienced by the enzyme upon cooling-induced loss of structural flexibility. Enzyme conformers may get trapped in regions of conformational space that lead to impaired catalysis. The coupled motion associated with the proposed precatalytic step could arguably be affected by the structural rigidification.

**Scheme 4:**
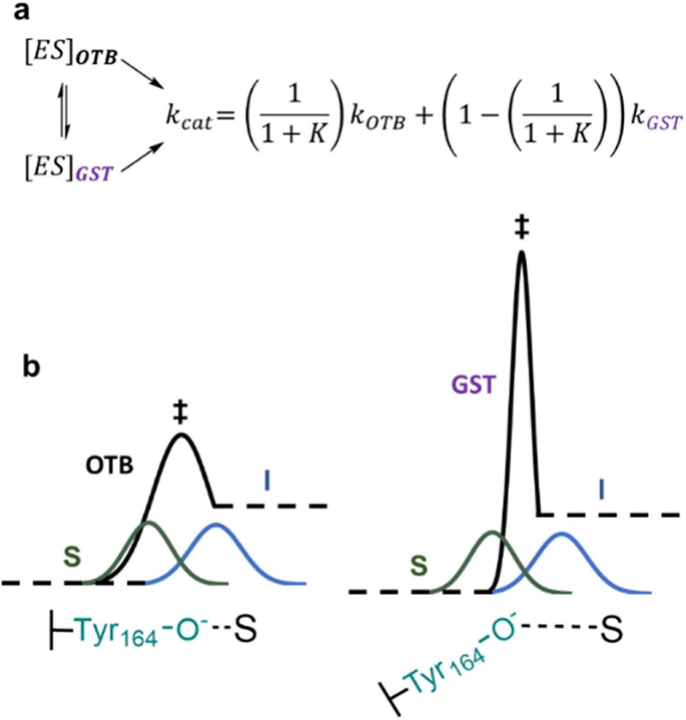
Two-State Equilibrium Model and Presumed Barrier Shapes with Wavefunction Overlap for the Epimerization of CDP-[2-^2^H]Glc and CDP-Glc (a) The conformational equilibrium between [ES]_OTB_ for “over-the-barrier” (OTB) high-temperature epimerization and [ES]_GST_ being the ground-state tunneling (GST) conformer formed at lower temperature is represented by *k*_cat_ as the sum of the weighted reaction rates *k*_OTB_ and *k*_GST_. Non-logarithmized experimental data was used for fitting and finding numerical solutions to the model proposed. For details, see Figure S9 and Materials and Methods. (b) Different enthalpic barrier heights/shapes for the OTB (left) and GST (right) conformer during substrate (S) oxidation to the 2-keto intermediate (I). Depictions of Tyr164 locked-on to the substrate in OTB and the moderate interaction in the GST conformer indicate the different levels of coupled motion in the respective substate. The equally overlapping substrate/intermediate wavefunctions (green; blue) allow for the possibility of tunneling controlled reactions in both conformers.

Not only might the coupled motion be slowed down upon the loss of protein flexibility, but it could also become less precise in the positioning for catalysis. Arrhenius profiles of the *k*_cat_ in the range 20–40 °C reveal *E*_A_ values for the reaction with the ^1^H and ^2^H substrates that differ by as much as −37 kJ mol^–1^. Explanation of the result requires the assumption of a heterogeneous population of enzyme molecules differing in their kinetic properties. The simplest case would be that of a two-state equilibrium between enzyme conformers that involve a different nature of the chemical step and thus turn over the product at different rates (Scheme 4). The *k*_cat_ is the sum of the individual rate constants weighted by the fraction of total enzyme present in the respective conformer. An observable *E*_A_ is then composite of the enthalpic barriers of the chemical steps in the two conformers, but Arrhenius plots are unlikely to be linear over a broad temperature range (see the profile for the ^2^H substrate in Figure 3).^107^ We hypothesize that conformational heterogeneity arises from the effect of low temperature on the conformational sampling achieved by coupled motion. In the proposed scenario, therefore, enzyme conformers featuring impaired positioning in the ground-state complex will experience a substantially elevated enthalpic barrier for the catalytic reaction. Partial loss of catalytic facilitation from proton abstraction to the active-site base can plausibly explain the effect. To undergo chemical conversion, such conformers will rely on a strongly increased contribution from quantum mechanical tunneling to the hydride transfer, compared to conformers that retain precise positioning to enable a lower enthalpic barrier. Lowering the temperature arguably shifts fractional occupation of enzyme conformers toward the ones with non-optimal positioning. The relative contribution to the observable *k*_cat_ from turnover of the two types of enzyme conformers will thus depend on temperature, with the reaction from the “tunneling conformer” gaining increased importance as the temperature is lowered. A simple mathematical model shown in Scheme 4 reasonably describes the overall trend of the data (Figure S9). Our two-state model is similar to that of Mulholland and co-workers^108^ to explain temperature-dependent KIEs in that it proposes one reactive conformation that proceeds by tunneling and one that passes over the barrier. The output of the proposed model predicts a drop to *E*_A_ = 0 for the combined enthalpic barriers in the low temperature regime, suggesting a tunneling controlled reaction and confirming the shift to a tunneling-ready conformer. The obtained equilibrium constant *K* (= [ES]_GST_/[ES]_OTB_) converges to ∼1, indicating fractional equality of the two conformers assumed.

The idea of temperature-dependent equilibration of substates in the conformational ensemble sampled by the *Ta*CPa2E enzyme–substrate complex was built on seminal studies of the thermostable ADH from *Bacillus stearothermophilus*.^23,25,44^ In this ADH, heat is required to promote transition from a conformationally restricted inactive state to a more flexible, active state.^25,43,55^*Ta*CPa2E differs from the ADH in that the impaired conformational state retains activity. As discussed above, this unique feature of the *Ta*CPa2E gives rise to an unprecedented temperature dependence of the catalytic rates and their associated KIEs. Important mechanistic insight derives from the evidence showing that the nature of the hydride transfer was fundamentally changed in consequence of the switch to the impaired conformational state at low temperature. Thus, coupled motions enabled by protein flexibility are linked to catalysis of the chemical step in the metal-independent enzymatic mechanism of alcohol oxidation.

### Dynamical Properties of Hydride Transfer in the High-Temperature Region

According to the proposed two-state model (Scheme 4), an increase in temperature to 40 °C and higher enables the coupled motion of *Ta*CPa2E to precision in positioning. Catalytically impaired conformers of the enzyme–substrate complex are thus re-equilibrated into the fully active region of the conformational space. From Arrhenius plots of the *k*_cat_, there is a +13 kJ mol^–1^ higher *E*_A_ for the reaction with ^2^H compared to the ^1^H substrate and the pre-factor *A*_H_/*A*_D_ ratio is 0.05. In the semiclassical interpretation of hydride transfer reactions, the *A*_H_/*A*_D_ ratio is indicative of the tunneling contribution to the chemical step.^33,108−110^ The *A*_H_/*A*_D_ ratio below the semiclassical limit of 0.5 suggests a moderate tunneling regime in which tunneling occurs primarily for the light isotope. The very small *A*_H_/*A*_D_ ratio found for the *Ta*CPa2E reaction implies a minimal tunneling contribution to the conversion of the deuterated CDP-glucose. The reaction can still occur, however, because the heat provided is sufficient. The inverse SKIE on *k*_cat_ with the CDP-[2-^2^H]Glc substrate suggests increased participation of tunneling to the reaction of the heavier isotope enabled by solvent deuteration. To expand the mechanistic interpretation beyond the limits of semiclassical transition state theory, we applied the Marcus-like full-tunneling model developed by Kohen and co-workers^13^ to describe our experimental data. Generally, models of this type extend the Marcus-theory of electron tunneling to hydrogen atom tunneling (involving proton tunneling) in which heavy-atom reorganization results in tunneling-ready states.^85,111,112^ The Kohen model further links the size and temperature dependence of the KIEs to a population distribution for fluctuations of the distance between donor and acceptor atoms.^13^ The data required fitting by a two-population distribution of DADs (Figure S7; fit with a single-population model was unsuccessful), with the long-distance population centered at a value of 3.31 ± 0.02 Å and a Gibbs free energy difference (Δ*G*) between the two populations of 17.24 ± 2.1 kJ mol^–1^. In Kohen’s model,^13^ the Δ*G* is relative to a short-distance population centered at DAD short enough for ^1^H and ^2^H to cross the dividing surface between the substrate and product with similar probabilities; hence, the KIE associated with the population is unity. Compared to the short-distance population, the second, long-distance population involves lower energy, decreased tunneling probability overall, and larger KIE. Temperature dependence of the KIE is thus explained from the temperature-dependent change in the relative populations present in thermal equilibrium. The Δ*G* for *Ta*CPa2E is among the largest seen for enzymes catalyzing hydride transfer. Only in one variant of DHFR (M42W-G121V) has a similar Δ*G* (18.42 kJ mol^–1^) been found.^30^ In both *Ta*CPa2E and DHFR variants, the unusually large Δ*G* probably reflects a very loose active site that involves substantial fluctuation in DAD for the tunneling ready state. Poor conformational reorganization can result in low-frequency DAD sampling and lead to a wide range of DADs exhibiting temperature dependence of their distribution. The long-distance populations of *Ta*CPa2E and the DHFR variant^13,30^ (DAD_L_ = 3.34 Å) center at similar values to DAD. Since wavefunctions for the substrate and product exhibit a scant overlap for DADs as long as these,^13^ the tunneling probability is low overall and in particular so for the heavy isotope, leading to a large KIE. An increasing temperature populates the short DAD from which the heavy isotope can also be transferred. The relative advantage of ^1^H tunneling from the longer DADs is thus lowered, and the KIE decreases in a strongly temperature-dependent manner.

### The Loose-Fit CDP-Glucose Substrate Resembles Mutations in Effect on the DAD Coordinate

A number of studies have found that within the limits of the experimental error, wild-type enzymes show temperature-independent KIEs on the hydride transfer.^9,34,113,114^ The behavior is generally explained by precise positioning of the substrate in the ground-state complex, leading to a narrow distribution of DADs at the tunneling-ready state of a well-preorganized protein conformation.^13,85^ A change in temperature will not cause a DAD population shift, and since both isotopes are affected similarly, the KIE change with temperature will be minimal. Mutations often promote a broader ensemble of conformations representing differently preorganized substates that can give rise to distinct populations along the DAD coordinate.^9,13^ Variants of DHFR with substitutions in (e.g., I14G^9^) or distal to the active site (e.g., M42W-G121V; M42W-F125M^7,30,115^) reveal the effect dramatically. As shown by phenomenological modeling, they display enhanced conformational flexibility, leading to increased population size and broadening of the DAD distributions. The experimental *E*_A_ is +13.8 kJ mol^–1^ (I14G),^9^ +15.5 kJ mol^–1^ (M42W-G121V),^30^ and +16.7 kJ mol^–1^ (M42W-F125W)^115^ larger for the reaction with the heavier (^3^H) isotope substrate. The KIE of *Ta*CPa2E probed with CDP-Glc exhibits a temperature dependence of similar magnitude to (even larger than the ones of) the DHFR variants. The observed Δ*E*_A_ is +13 kJ mol^–1^ but one must take into account that the ^2^H KIE instead of the ^3^H KIE was measured for the epimerase, and the zero-point energy of the ^2^H-C bond lies +2.26 kJ mol^–1^ higher than that of the ^3^H–C bond. Considering that the *Ta*CPa2E used here is the wild-type enzyme, the strongly inflated Δ*E*_A_ warrants further discussion. Its molecular interpretation is that the enzymatic oxidation of CDP-Glc involves extensive dynamical sampling of DADs suitable for tunneling. Requirement for the enzyme to explore a large conformational space in order to promote catalysis is consistent with earlier reported evidence by computational docking. The native 3,6-dideoxygenated substrate CDP-paratose (Scheme 1) fits snugly into the preorganized binding pocket of the *Ta*CPa2E. The CDP-Glc can be accommodated only with substantial structural rearrangements of protein residues, to create sufficient space for the 3-OH and 6-OH sugar substrate groups.^72^ Temperature dependence of the KIE for non-preferred substrates has only been scantly studied with enzymes. Proton transfer by methylamine dehydrogenase involves a KIE that is temperature-independent with the native substrate methylamine but temperature-dependent (Δ*E*_A_ = +8.4 kJ mol^–1^; *A*_H_/*A*_D_ = 0.57) with the slow substrate ethanolamine.^116^ The increased requirement of active site rearrangements for positioning bulky substrates has been recognized in protein KIE studies of alcohol dehydrogenase.^117^ The current results emphasize the mechanistic utility of a structurally loose-fit, slowly reacting substrate to probe dynamical features of the chemical step of hydride transfer in enzymatic sugar nucleotide epimerization.

In summary, temperature dependence of the primary deuterium KIE for C2 epimerization of CDP-glucose reveals protein conformational selection linked to catalysis by the thermophilic *Ta*CPa2E. In the multistep catalytic process of the epimerase, the instigating hydride abstraction by enzyme-NAD^+^ is shown to be rate-limiting. A hierarchy of temperature dependent protein motions is necessary for optimal catalysis to the C–H bond cleavage. Evidence suggests that coupled motions achieve precise positioning of the substrate so that partial proton abstraction can effectively initiate the bond breaking. Protein rigidity at low temperature (≤ 40 °C) interferes with conformational sampling by the coupled motion, thus giving rise to catalytically impaired enzyme conformers that lack suitable activation of the proton transfer. Quantum mechanical tunneling associated with the C–H bond cleavage identifies protein dynamics necessary in catalysis. To promote the chemical step in the high temperature region, the epimerase requires extensive dynamical sampling of DADs suitable for tunneling. Due to the high and narrow enthalpic barrier for conversion of the conformationally restricted enzyme substates, the epimerase catalysis is tunneling controlled in the low temperature region. Overall, the contributions of protein dynamics to catalysis of C–H bond cleavage by *Ta*CPa2E show important analogies to ADHs and DHFRs catalyzing the same bond breaking in simpler enzymatic transformations. A loose-fit substrate (in *Ta*CPa2E) resembles structural variants of ADHs and DHFRs in its requirement for extensive dynamical sampling to balance conformational flexibility and catalytic efficiency. Protein motions associated with intermediate rotation and reduction of the epimerase reaction are not revealed at the steady state.

## Materials and Methods

### Materials

Nucleotides and ^1^H-glucose were from Carbosynth (Compton, Berkshire, U.K.). ^2^H-glucose was from Sigma-Aldrich (Vienna, Austria). Deuterium oxide (99.96% ^2^*H*) was from Euriso-Top (Saint-Aubin Cedex, France). All other chemicals and reagents were of the highest available purity. *E. coli* BL21(DE3) competent cells were prepared in-house. DpnI and Q5 High-Fidelity DNA polymerases were from New England Biolabs (Frankfurt am Main, Germany). For plasmid DNA isolation, a GeneJET Plasmid Miniprep Kit (Thermo Scientific; Waltham, MA, USA) was used.

### Enzymes

The *Ta*CPa2E_Y164F variant was prepared via a modified QuikChange protocol. For PCRs, 20 ng of plasmid DNA (template) and 0.2 μM of forward or reverse primer in a total reaction volume of 50 μL were used. DNA amplification was carried out with a Q5 DNA polymerase. Primer sequences used for mutagenesis are shown in Table S2. *Ta*CPa2E wild-type and the Y164F variant were expressed in *E. coli* BL21 (DE3) cells harboring a pET21a expression vector with the respective gene. Enzyme purification was performed using the C-terminal His-tag. Separate His-tag columns were used for the wild-type *Ta*CPa2E and Y164F variant to traces of one enzyme carried over into the other’s preparation. Molecular mass and purity of the proteins were confirmed by SDS-PAGE (see Figure S10 for the Y164F variant). Detailed information on expression and purification conditions is given elsewhere.^72^

### Substrate Synthesis and Isolation

Details of the enzymatic synthesis of CDP-Glc can be found elsewhere.^72^ The synthesis of CDP-[2-^2^H]Glc was identical to the synthesis of the unlabeled counterpart (Scheme S1). Briefly, anomeric phosphorylation was carried out using *N*-acetylhexosamine 1-kinase, ATP, MgCl_2_, and [2-^2^H]glucose in MOPS buffer (100 mM, pH 7.5). The reaction mixture was incubated at 30 °C until ATP was fully depleted. Nucleotidyl transfer from CTP to [2-^2^H]glucose-1-phosphate was catalyzed by UDP-glucose pyrophosphorylase. An inorganic pyrophosphatase was used for pyrophosphate removal. Product isolation was performed as described elsewhere.^72^ Both CDP-[2-^2^H]Glc and CDP-Glc were obtained in high purity (≥ 95%) and in amounts of 10–20 mg. The identity of CDP-[2-^2^H]Glc was confirmed by HPLC and ^1^H-NMR (Figures S11 and S12). The ^2^H content at C2 was 95% or greater.

### Determination of Kinetic Isotope Effects

#### Primary Kinetic Isotope Effects

Primary KIEs on the *k*_cat_ were determined by carrying out epimerization reactions of CDP-[2-^2^H]Glc and CDP-Glc (4.00 mM) for 15 min in MOPS buffer (100 mM, pH 7.5) using purified *Ta*CPa2E (1.0 mg mL^–1^; 25.4 μM) at varying temperatures (20–80 °C) in a total reaction volume of 60 μL. CDP-Glc/CDP-[2-^2^H]Glc (15 μL; 16 mM stock) were added to 45 μL of prepared enzyme solution (10 μL of 6.0 mg mL^–1^*Ta*CPa2E + 35 μL buffer). The enzyme solution was equilibrated for 5 min at the desired reaction temperature in a non-agitated thermomixer comfort (Eppendorf AG, Hamburg, Germany). Potential temperature-induced shifts in pH values were ruled out as no changes prior to, or after completion of, the reaction were detected. Samples were taken at 0, 5, and 15 min, and the reaction was quenched by adding 5 μL of sample to 25 μL of doubly distilled water and 30 μL of methanol (1/1 ratio water/methanol). Mixtures were vortexed (10 s), centrifuged for 45 min at 21130*g*, and analyzed on HPLC. Substrate and enzyme concentrations were kept constant over the entire temperature range to assure the highest reliability and reproducibility of the data. Conversions to the respective D-*manno*-configured isotopes (0.2–10%) were within the initial rate period (see Figure S2 for 60 °C reaction), which were calculated from the linear segment of the time course by means of dividing the slope of the linear fit (mM min^–1^) by the enzyme concentration (mg mL^–1^). The apparent *k*_cat_ values (min^–1^) were calculated from the initial rate (= μmol (min mg_protein_)^−1^) with the molecular mass of the functional enzyme monomer (*Ta*CPa2E_WT: 39362 g mol^–1^). Substrate saturating conditions were achieved by using substrate concentrations 12.5-fold above the *K*_M_ value (0.32 ± 0.02 mM) at 60 °C. It was further ensured that the increase in the substrate concentration by 0.5 and 1.0 mM did not change the rates obtained at 4.0 mM with limits of error (± 5%). Repeated measurements (*N* ≥ 4) at a single saturating substrate concentration gave *k*_cat_ values determined with high precision. The KIEs (Table S1) were obtained as the ratio of the *k*_cat_ for enzymatic reaction with the light (^1^H) and heavy (^2^H) isotope.

The KIE on the *k*_cat_/*K*_M_ was determined for reactions at 20, 60, and 80 °C. Initial rates were acquired with *Ta*CPa2E (1.0 mg mL^–1^; 25.4 μM) at varying substrate concentration (0.1–5.0 mM) of both CDP-Glc isotopes (Table 2 and Figure S3) in MOPS-H_2_O buffer (100 mM, pH 7.5). Samples were taken in 2.5 min intervals and quenched by methanol (1/1 ratio water/methanol). Calculation of the specific rate (*V*/[E], min^–1^) was based on linear correlations of the substrate consumed and the product released within the reaction time. For further experimental details on the kinetic characterization of the enzymatic reaction with CDP-Glc, see ref (72). SigmaPlot 10.0 (Systat Software, Inc.) was used for fitting eq 6 to the experimental data.
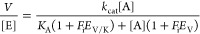
6

In eq 6, [A] is the molar concentration of CDP-Glc, *K*_A_ is the Michaelis constant, *F*_i_ is the fraction of deuterium in the substrate (0.95), and *E*_V_ and *E*_V/K_ are KIEs minus 1 on *k*_cat_ and *k*_cat_/*K*_M._^118^ All experiments were performed in triplicate (S.D. shown in Figure S3), and mean values were used for fitting. Note that the KIEs on *k*_cat_ determined from fits of eq 6 to the data were in excellent agreement (± 5%) with the corresponding KIEs determined from (repeated) measurements at a single saturating substrate concentration.

#### Solvent Kinetic Isotope Effects

*Ta*CPa2E (48.0 mg mL^–1^; 1.2 mM) was re-buffered to MOPS-^2^H_2_O (100 mM, p^2^H 7.5) by centrifugation (2100*g*, 10 °C) using 0.5 mL VivaSpin tubes (10 kDa cutoff) until a dilution factor of 500 was reached. The enzyme was further incubated for 0.25 or 4.5 h at 4 °C. The incubation in ^2^H2O solvent did not cause loss of enzyme activity (measured in ^1^H2O). The substrate preparation, reaction setup, and analysis followed the procedure as described for the determination of primary KIE. CDP-[2-^2^H]Glc stocks were prepared in MOPS-^2^H_2_O (100 mM). Reactions in MOPS-^1^H_2_O buffer were carried out with purified *Ta*CPa2E (48.0 mg mL^–1^; 1.2 mM) stored in MOPS-^1^H_2_O (100 mM). SKIEs were determined at pL (L = ^1^H or ^2^H) 7.0, 7.5, and 8.0 at 60 °C and pL 7.5 at 20 °C. Desired pL values were obtained by adjusting MOPS-^2^H_2_O (100 mM) buffers with NaOH dissolved in ^2^H_2_O. The p^2^H was obtained as a pH meter reading of +0.4. MOPS-^1^H_2_O (100 mM, pH 7.5) containing 9% v/v glycerol was used as viscosity control.^86,103^ It was also shown in kinetic measurements at 60 °C that the time of pre-incubation in ^2^H_2_O (0.25 h, 4.5 h) had no effect on the observable SKIEs within limits of error. All measurements were therefore done with enzyme exchanged in ^2^H_2_O solvent for 4.5 h. Experiments were performed in triplicate. SKIEs (Table 3) were obtained as the ratio of the *k*_cat_ for the enzymatic reaction in ^1^H_2_O and ^2^H_2_O. Superscript D_2_O (^D_2_O^*k*_cat_) is used to indicate the SKIE.

### Determination of Enzyme-Bound NADH/NAD^+^ in the Steady State

Purified *Ta*CPa2E (1.0 mg mL^–1^; 25.4 μM) and CDP-Glc (4.00 mM) in a final volume of 1 mL were reacted for 15 min in MOPS-^1^H_2_O/^2^H_2_O buffer (100 mM, pL 7.5) at 60 °C without agitation (thermomixer comfort; Eppendorf AG, Hamburg, Germany). The reaction was started by adding 200 μL of CDP-Glc (20 mM stock) to 800 μL of equilibrated (5 min, 60 °C) enzyme solution (1.25 mg mL^–1^ stock). Control reactions lacking CDP-Glc were prepared using 200 μL of MOPS-^1^H_2_O/^2^H_2_O buffer (100 mM, pL 7.5) and 800 μL of enzyme solution. The reaction/control was quenched by adding ice-cold acidified MOPS-^1^H_2_O/^2^H_2_O buffer (100 mM, pL 1.9) in a 1/1 ratio, resulting in a final pL of 5.5 and retaining the enzymes’ solubility while terminating the enzymatic activity. The reaction/control was transferred into ice-cold 5 mL VivaSpin tubes (10 kDa MWCO) and centrifuged at 10 °C (2880*g*) to a final volume of 0.5 mL. The concentrated reaction/control solution was washed twice by adding 4.5 mL of acidified buffer and repeating the centrifugation step. The flowthroughs were analyzed on HPLC to assure that the reaction equilibrium remained unchanged over the washing process (see Figure S5). The reaction/control (0.5 mL) was transferred into ice-cold 0.5 mL VivaSpin tubes (10 kDa MWCO) and concentrated to a final volume of 50 μL (2880 g, 10 °C), followed by determination of the protein concentrations on a Nanodrop at 280 nm using the *Ta*CPa2E-specific extinction coefficient ε = 16.27 M^–1^ cm^–1^ (ProtParam tool, Expasy, Swiss Bioinformatics Resource Portal). Protein precipitation was initiated by adding MeOH (50 μL) to the reaction/control and incubation for 2.5 h at 30 °C (no agitation). Afterward, the samples were centrifuged for 45 min at 21130*g*. The supernatant was withdrawn, and the precipitated enzyme was re-suspended in 50 μL of 6 M urea followed by HPLC analysis. Additionally, NADH stability was tested by subjecting 0.1 mM NADH dissolved in MOPS buffer (100 mM, pH 7.5) to the same procedure as described above. No degradation of the coenzyme was found. Experiments were performed in quadruplicate. Note that based on experiments (*N* = 8) aimed at showing the reproducibility of the coenzyme extraction method, enzyme-bound NAD^+^ could not be extracted from *Ta*CPa2E reliably (reproducibility ≥28%), whereas extraction of the less tightly-bound NADH proved to be highly dependable (reproducibility of ≥86%). An NADH calibration curve (Figure S5) was used for calculating the concentration of the released NADH (μM). The amount of enzyme-bound NADH (%) was determined by dividing the protein concentration (μM) measured prior to enzyme denaturation (∼96% of the original amount) into the released NADH concentration (μM). Calculation of the amount of CDP-mannose and CDP-glucose (μM) associated with the denatured enzyme (μM) was based on calibration curves for both compounds.

### Analytics

#### HPLC

A Shimadzu Prominence HPLC-UV system equipped with a Kinetex C18 analytical HPLC column was used. Injection volumes were between 5 and 30 μL. For KIE experiments, UV detection at 271 nm using a Kinetex C18 column (150 × 4.6 mm, 5 μm EVO C18 100 Å; Phenomenex, Aschaffenburg, Germany) and an isocratic flow (1 mL/min) at 40 °C with a mobile phase composed of 20 mM potassium phosphate buffer (pH 5.9) containing 40 mM tetrabutylammonium bromide (98%; solvent A) and methanol (2%; solvent B) was applied. HPLC analysis in the course of the rapid-quench assay was performed using a Kinetex C18 column (50 × 4.6 mm, 5 μm 100 Å) and an isocratic flow (2 mL/min) with 40 mM tetrabutylammonium bromide (95%; solvent A) and acetonitrile (5%; solvent B) in 20 mM potassium phosphate buffer (pH 5.9).

#### ^1^H-NMR Analysis

The reaction mixture prepared for in situ proton NMR analysis contained 58.4 μM (2.3 mg/mL) *Ta*CPa2E and 2.00 mM CDP-[2-^2^H]Glc in ^2^H_2_O buffer (50 mM K_2_HPO_4_/KH_2_PO_4_, p^2^H 7.5; p^2^H = pH meter reading +0.4). The data was acquired at 60 °C on a Varian INOVA 500-MHz NMR spectrometer (Agilent Technologies, Santa Clara, California, USA) in 10 min intervals starting from enzyme addition using VNMRJ 2.2D software. ^1^H-NMR spectra (499.98 MHz) were recorded with pre-saturation of the water signal by a shaped pulse on a 5 mm indirect detection PFG probe. Spectra were analyzed using MestReNova 16.0 (Mestrelab Research, S.L.). In situ proton NMR analysis of *Ta*CPa2E/CDP-Glc was described elsewhere.^72^

### Bioinformatic and Computational Tools

#### Two-State Equilibrium Model

Data fitting was performed using Microsoft Excel’s Solver add-in and the GRG Nonlinear solving method. Constraint precision was set to 10^–6^ and convergence to 10^–4^. The sum of the relative errors squared was minimized. A unique solution was obtained by multiple fitting events to eqs 7a and 7b.

Fit 1:
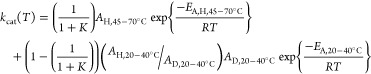
7a

Fit 2:
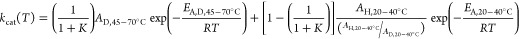
7b

Fitting parameters, constraints, and model outputs including the equilibrium constant *K* (= [ES]_GST_/[ES]_OTB_) are presented in Figure S9.

#### Calculation of Donor–Acceptor Distances

DAD calculations were performed applying the program provided and developed by Roston et al.^13^ at http://chemmath.chem.uiowa.edu/webMathematica/kohen/marcuslikemodel.html.

#### Enzyme/Substrate Structure model

The PyMOL Molecular Graphics System (Open-Source, Schrödinger, LLC) was used for depicting donor–acceptor distances in TaCPa2E. Structure modeling and ligand docking conducted for Figure S8 were described elsewhere.^72^

## Supporting Information Available

Full ^1^H NMR spectra, KIE values, HPLC-chromatograms, DAD calculation, PCR method, model output and details of the synthesis and analysis of CDP-[2-^2^H]Glc; KIE values obtained for reacting *Ta*CPa2E with CDP-Glc or CDP-[2-^2^H]Glc in the temperature range of 293.15–353.15 K (Table S1); DNA primer sequence for the *Ta*CPa2E_Y164F variant (Table S2) and description of the PCR method; determination of the noncompetitive KIE using CDP-[2-^2^H]Glc or CDP-Glc (Figure S1); exemplified comparison of the initial rates for the epimerization of CDP-Glc isotopes (Figure S2); kinetic data used for determination of ^D^*k*_cat_/*K*_M_ or ^D_2_O^*k*_cat_/*K*_M_ at different temperatures (Figure S3); complementing full spectrum of in situ ^1^H-NMR measurements applying *Ta*CPa2E and CDP-[2-^2^H]Glc or CDP-Glc (Figure S4); NADH quantification and HPLC analysis of flowthrough and supernatant to assure an unchanged reaction equilibrium of CDP-Glc/CDP-Man in the rapid-quench assay (Figure S5); determination of the reaction equilibrium for *Ta*CPa2E with CDP-[2-^2^H]Glc (Figure S6); DAD calculation for *Ta*CPa2E obtained by fitting hydrogen/deuterium (H/D) KIEs against the inverse temperature (Figure S7); donor–acceptor distance in the structure model of *Ta*CPa2E (Figure S8); two-state equilibrium model output with fitting parameters and set constraint values (Figure S9); SDS-PAGE result of the purified *Ta*CPa2E_Y164F variant (Figure S10); HPLC chromatogram (Figure S11) and ^1^H-NMR spectrum of CDP-[2-^2^H]Glc (Figure S12); enzymatic synthesis of CDP-[2-^2^H]Glc (Scheme S1) with short description of the methods used (PDF)

## Abbreviations

*Ta*CPa2E: *Thermodesulfatator atlanticus* CDP-paratose 2-epimerase
DAD: donor–acceptor distance
KIE: kinetic isotope effect
CDP-Glc: CDP-α-d-glucose
CDP-Man: CDP-α-d-mannose
CDP-[2-^2^H]Glc: CDP-[2-^2^H]α-d-glucose
CDP-[2-^2^H]Man: CDP-[2-^2^H]α-d-mannose

## References

[ref1] Agarwal P. K. A Biophysical Perspective on Enzyme Catalysis. Biochemistry 2019, 58, 438–449. 10.1021/acs.biochem.8b01004. 30507164 PMC6386455

[ref2] Hammes-Schiffer S.; Benkovic S. J. Relating Protein Motion to Catalysis. Annu. Rev. Biochem. 2006, 75, 519–541. 10.1146/annurev.biochem.75.103004.142800. 16756501

[ref3] Cheatum C. M. Low-Frequency Protein Motions Coupled to Catalytic Sites. Annu. Rev. Phys. Chem. 2020, 71, 267–288. 10.1146/annurev-physchem-050317-014308. 32312192

[ref4] Nashine V. C.; Hammes-Schiffer S.; Benkovic S. J. Coupled Motions in Enzyme Catalysis. Curr. Opin. Chem. Biol. 2010, 14, 644–651. 10.1016/j.cbpa.2010.07.020. 20729130 PMC2953590

[ref5] Nagel Z. D.; Klinman J. P. Update 1 of: Tunneling and Dynamics in Enzymatic Hydride Transfer. Chem. Rev. 2010, 110, 41–67. 10.1021/cr1001035. PMC4067601 21141912

[ref6] Sikorski R. S.; Wang L.; Markham K. A.; Rajagopalan P. T. R.; Benkovic S. J.; Kohen A. Tunneling and Coupled Motion in the *Escherichia coli* Dihydrofolate Reductase Catalysis. J. Am. Chem. Soc. 2004, 126, 4778–4779. 10.1021/ja031683w. 15080672

[ref7] Singh P.; Abeysinghe T.; Kohen A. Linking Protein Motion to Enzyme Catalysis. Molecules 2015, 20, 1192–1209. 10.3390/molecules20011192. 25591120 PMC4341894

[ref8] Klinman J. P. Dynamically Achieved Active Site Precision in Enzyme Catalysis. Acc. Chem. Res. 2015, 48, 449–456. 10.1021/ar5003347. 25539048 PMC4334267

[ref9] Stojković V.; Perissinotti L. L.; Willmer D.; Benkovic S. J.; Kohen A. Effects of the Donor-Acceptor Distance and Dynamics on Hydride Tunneling in the Dihydrofolate Reductase Catalyzed Reaction. J. Am. Chem. Soc. 2012, 134, 1738–1745. 10.1021/ja209425w. 22171795 PMC4341912

[ref10] Narayanan C.; Bernard D.; Doucet N. Role of Conformational Motions in Enzyme Function: Selected Methodologies and Case Studies. Catalysts 2016, 6, 81 10.3390/catal6060081. 28367322 PMC5375114

[ref11] Benkovic S. J.; Fierke C. A.; Naylor A. M. Insights into Enzyme Function from Studies on Mutants of Dihydrofolate Reductase. Science 1988, 239, 1105–1110. 10.1126/science.3125607. 3125607

[ref12] Benkovic S. J.; Hammes G. G.; Hammes-Schiffer S. Free-Energy Landscape of Enzyme Catalysis. Biochemistry 2008, 47, 3317–3321. 10.1021/bi800049z. 18298083

[ref13] Roston D.; Cheatum C. M.; Kohen A. Hydrogen Donor-Acceptor Fluctuations from Kinetic Isotope Effects: A Phenomenological Model. Biochemistry 2012, 51, 6860–6870. 10.1021/bi300613e. 22857146 PMC3448806

[ref14] Hammes-Schiffer S. Hydrogen Tunneling and Protein Motion in Enzyme Reactions. Acc. Chem. Res. 2006, 39, 93–100. 10.1021/ar040199a. 16489728

[ref15] Wong K. F.; Selzer T.; Benkovic S. J.; Hammes-Schiffer S. Impact of Distal Mutations on the Network of Coupled Motions Correlated to Hydride Transfer in Dihydrofolate Reductase. Proc. Natl. Acad. Sci. U. S. A. 2005, 102, 6807–6812. 10.1073/pnas.0408343102. 15811945 PMC1100751

[ref16] Benkovic S. J.; Hammes-Schiffer S. Enzyme Motions Inside and Out. Science 2006, 312, 208–209. 10.1126/science.1127654. 16614206

[ref17] Silva R. G.; Murkin A. S.; Schramm V. L. Femtosecond Dynamics Coupled to Chemical Barrier Crossing in a Born-Oppenheimer Enzyme. Proc. Natl. Acad. Sci. U. S. A. 2011, 108, 18661–18665. 10.1073/pnas.1114900108. 22065757 PMC3219149

[ref18] Zoi I.; Suarez J.; Antoniou D.; Cameron S. A.; Schramm V. L.; Schwartz S. D. Modulating Enzyme Catalysis through Mutations Designed to Alter Rapid Protein Dynamics. J. Am. Chem. Soc. 2016, 138, 3403–3409. 10.1021/jacs.5b12551. 26927977 PMC4794390

[ref19] Schramm V. L.; Schwartz S. D. Promoting Vibrations and the Function of Enzymes. Emerging Theoretical and Experimental Convergence. Biochemistry 2018, 57, 3299–3308. 10.1021/acs.biochem.8b00201. 29608286 PMC6008225

[ref20] Otten R.; Pádua R. A. P.; Bunzel H. A.; Nguyen V.; Pitsawong W.; Patterson M.; Sui S.; Perry S. L.; Cohen A. E.; Hilvert D.; Kern D. How Directed Evolution Reshapes the Energy Landscape in an Enzyme to Boost Catalysis. Science 2020, 370, 1442–1446. 10.1126/science.abd3623. 33214289 PMC9616100

[ref21] Gardner J. M.; Biler M.; Risso V. A.; Sanchez-Ruiz J. M.; Kamerlin S. C. L. Manipulating Conformational Dynamics To Repurpose Ancient Proteins for Modern Catalytic Functions. ACS Catal. 2020, 10, 4863–4870. 10.1021/acscatal.0c00722.

[ref22] Crean R. M.; Gardner J. M.; Kamerlin S. C. L. Harnessing Conformational Plasticity to Generate Designer Enzymes. J. Am. Chem. Soc. 2020, 142, 11324–11342. 10.1021/jacs.0c04924. 32496764 PMC7467679

[ref23] Kohen A.; Klinman J. P. Protein Flexibility Correlates with Degree of Hydrogen Tunneling in Thermophilic and Mesophilic Alcohol Dehydrogenases. J. Am. Chem. Soc. 2000, 122, 10738–10739. 10.1021/ja002229k.

[ref24] Roston D.; Kohen A. Elusive Transition State of Alcohol Dehydrogenase Unveiled. Proc. Natl. Acad. Sci. U. S. A. 2010, 107, 9572–9577. 10.1073/pnas.1000931107. 20457944 PMC2906880

[ref25] Nagel Z. D.; Dong M.; Bahnson B. J.; Klinman J. P. Impaired Protein Conformational Landscapes as Revealed in Anomalous Arrhenius Prefactors. Proc. Natl. Acad. Sci. U. S. A. 2011, 108, 10520–10525. 10.1073/pnas.1104989108. 21670258 PMC3127881

[ref26] Alhambra C.; Corchado J. C.; Sánchez M. L.; Gao J.; Truhlar D. G. Quantum Dynamics of Hydride Transfer in Enzyme Catalysis. J. Am. Chem. Soc. 2000, 122, 8197–8203. 10.1021/ja001476l.

[ref27] Sen A.; Kohen A. Enzymatic Tunneling and Kinetic Isotope Effects: Chemistry at the Crossroads. J. Phys. Org. Chem. 2010, 23, 613–619. 10.1002/poc.1633.

[ref28] Singh P.; Vandemeulebroucke A.; Li J.; Schulenburg C.; Fortunato G.; Kohen A.; Hilvert D.; Cheatum C. M. Evolution of the Chemical Step in Enzyme Catalysis. ACS Catal. 2021, 11, 6726–6732. 10.1021/acscatal.1c00442.

[ref29] Pu J.; Ma S.; Gao J.; Truhlar D. G. Small Temperature Dependence of the Kinetic Isotope Effect for the Hydride Transfer Reaction Catalyzed by *Escherichia coli* Dihydrofolate Reductase. J. Phys. Chem. B 2005, 109, 8551–8556. 10.1021/jp051184c. 16852008 PMC4476250

[ref30] Wang L.; Goodey N. M.; Benkovic S. J.; Kohen A. Coordinated Effects of Distal Mutations on Environmentally Coupled Tunneling in Dihydrofolate Reductase. Proc. Natl. Acad. Sci. U. S. A. 2006, 103, 15753–15758. 10.1073/pnas.0606976103. 17032759 PMC1635075

[ref31] Wang Z.; Antoniou D.; Schwartz S. D.; Schramm V. L. Hydride Transfer in DHFR by Transition Path Sampling, Kinetic Isotope Effects, and Heavy Enzyme Studies. Biochemistry 2016, 55, 157–166. 10.1021/acs.biochem.5b01241. 26652185 PMC4752833

[ref32] Abeysinghe T.; Hong B.; Wang Z.; Kohen A. Preserved Hydride Transfer Mechanism in Evolutionarily Divergent Thymidylate Synthases. Curr. Top. Biochem. Res. 2016, 17, 19–30. 28018055 PMC5172458

[ref33] Roston D.; Islam Z.; Kohen A. Isotope Effects as Probes for Enzyme Catalyzed Hydrogen-Transfer Reactions. Molecules 2013, 18, 5543–5567. 10.3390/molecules18055543. 23673528 PMC4342783

[ref34] Pagano P.; Guo Q.; Ranasinghe C.; Schroeder E.; Robben K.; Häse F.; Ye H.; Wickersham K.; Aspuru-Guzik A.; Major D. T.; Gakhar L.; Kohen A.; Cheatum C. M. Oscillatory Active-Site Motions Correlate with Kinetic Isotope Effects in Formate Dehydrogenase. ACS Catal. 2019, 9, 11199–11206. 10.1021/acscatal.9b03345. 33996196 PMC8118594

[ref35] Antoniou D.; Schwartz S. D. Role of Protein Motions in Catalysis by Formate Dehydrogenase. J. Phys. Chem. B 2020, 124, 9483–9489. 10.1021/acs.jpcb.0c05725. 33064490 PMC7697370

[ref36] Pudney C. R.; Johannissen L. O.; Sutcliffe M. J.; Hay S.; Scrutton N. S. Direct Analysis of Donor–Acceptor Distance and Relationship to Isotope Effects and the Force Constant for Barrier Compression in Enzymatic H-Tunneling Reactions. J. Am. Chem. Soc. 2010, 132, 11329–11335. 10.1021/ja1048048. 20698699

[ref37] Basran J.; Harris R. J.; Sutcliffe M. J.; Scrutton N. S. H-Tunneling in the Multiple H-Transfers of the Catalytic Cycle of Morphinone Reductase and in the Reductive Half-Reaction of the Homologous Pentaerythritol Tetranitrate Reductase. J. Biol. Chem. 2003, 278, 43973–43982. 10.1074/jbc.M305983200. 12941965

[ref38] Hardman S. J. O.; Pudney C. R.; Hay S.; Scrutton N. S. Excited State Dynamics Can Be Used to Probe Donor-Acceptor Distances for H-Tunneling Reactions Catalyzed by Flavoproteins. Biophys. J. 2013, 105, 2549–2558. 10.1016/j.bpj.2013.10.015. 24314085 PMC3853081

[ref39] Chen X.; Schwartz S. D. Multiple Reaction Pathways in the Morphinone Reductase-Catalyzed Hydride Transfer Reaction. ACS Omega 2020, 5, 23468–23480. 10.1021/acsomega.0c03472. 32954200 PMC7496013

[ref40] Klinman J. P.; Offenbacher A. R.; Hu S. Origins of Enzyme Catalysis: Experimental Findings for C–H Activation, New Models, and Their Relevance to Prevailing Theoretical Constructs. J. Am. Chem. Soc. 2017, 139, 18409–18427. 10.1021/jacs.7b08418. 29244501 PMC5812730

[ref41] Knapp M. J.; Rickert K.; Klinman J. P. Temperature-Dependent Isotope Effects in Soybean Lipoxygenase-1: Correlating Hydrogen Tunneling with Protein Dynamics. J. Am. Chem. Soc. 2002, 124, 3865–3874. 10.1021/ja012205t. 11942823

[ref42] Rubach J. K.; Plapp B. V. Amino Acid Residues in the Nicotinamide Binding Site Contribute to Catalysis by Horse Liver Alcohol Dehydrogenase. Biochemistry 2003, 42, 2907–2915. 10.1021/bi0272656. 12627956

[ref43] Kohen A.; Cannio R.; Bartolucci S.; Klinman J. P. Enzyme Dynamics and Hydrogen Tunnelling in a Thermophilic Alcohol Dehydrogenase. Nature 1999, 399, 496–499. 10.1038/20981. 10365965

[ref44] Bruice Z.; Bruice T. C. Temperature-Dependent Structure of the E·S Complex of *Bacillus stearothermophilus* Alcohol Dehydrogenase. Biochemistry 2007, 46, 837–843. 10.1021/bi062110+. 17223705

[ref45] Caratzoulas S.; Mincer J. S.; Schwartz S. D. Identification of a Protein-Promoting Vibration in the Reaction Catalyzed by Horse Liver Alcohol Dehydrogenase. J. Am. Chem. Soc. 2002, 124, 3270–3276. 10.1021/ja017146y. 11916410

[ref46] Liang Z.-X.; Tsigos I.; Lee T.; Bouriotis V.; Resing K. A.; Ahn N. G.; Klinman J. P. Evidence for Increased Local Flexibility in Psychrophilic Alcohol Dehydrogenase Relative to Its Thermophilic Homologue. Biochemistry 2004, 43, 14676–14683. 10.1021/bi049004x. 15544338

[ref47] Kim K.; Plapp B. V. Substitutions of Amino Acid Residues in the Substrate Binding Site of Horse Liver Alcohol Dehydrogenase Have Small Effects on the Structures but Significantly Affect Catalysis of Hydrogen Transfer. Biochemistry 2020, 59, 862–879. 10.1021/acs.biochem.9b01074. 31994873

[ref48] Knapp M. J.; Klinman J. P. Environmentally Coupled Hydrogen Tunneling. Eur. J. Biochem. 2002, 269, 3113–3121. 10.1046/j.1432-1033.2002.03022.x. 12084051

[ref49] Tresadern G.; McNamara J. P.; Mohr M.; Wang H.; Burton N. A.; Hillier I. H. Calculations of Hydrogen Tunnelling and Enzyme Catalysis: A Comparison of Liver Alcohol Dehydrogenase, Methylamine Dehydrogenase and Soybean Lipoxygenase. Chem. Phys. Lett. 2002, 358, 489–494. 10.1016/S0009-2614(02)00654-1.

[ref50] Rucker J.; Klinman J. P. Computational Study of Tunneling and Coupled Motion in Alcohol Dehydrogenase-Catalyzed Reactions: Implication for Measured Hydrogen and Carbon Isotope Effects. J. Am. Chem. Soc. 1999, 121, 1997–2006. 10.1021/ja9824425.

[ref51] Hammann B.; Razzaghi M.; Kashefolgheta S.; Lu Y. Imbalanced Tunneling Ready States in Alcohol Dehydrogenase Model Reactions: Rehybridization Lags behind H-Tunneling. Chem. Commun. 2012, 48, 11337 10.1039/c2cc36110h. 23082319

[ref52] Antoniou D.; Caratzoulas S.; Kalyanaraman C.; Mincer J. S.; Schwartz S. D. Barrier Passage and Protein Dynamics in Enzymatically Catalyzed Reactions. Eur. J. Biochem. 2002, 269, 3103–3112. 10.1046/j.1432-1033.2002.03021.x. 12084050

[ref53] Hay S.; Scrutton N. S. Good Vibrations in Enzyme-Catalysed Reactions. Nat. Chem. 2012, 4, 161–168. 10.1038/nchem.1223. 22354429

[ref54] Benkovic S. J.; Hammes-Schiffer S. A Perspective on Enzyme Catalysis. Science 2003, 301, 1196–1202. 10.1126/science.1085515. 12947189

[ref55] Liang Z.-X.; Lee T.; Resing K. A.; Ahn N. G.; Klinman J. P. Thermal-Activated Protein Mobility and Its Correlation with Catalysis in Thermophilic Alcohol Dehydrogenase. Proc. Natl. Acad. Sci. U. S. A. 2004, 101, 9556–9561. 10.1073/pnas.0403337101. 15210941 PMC470713

[ref56] Plapp B. V. Conformational Changes and Catalysis by Alcohol Dehydrogenase. Arch. Biochem. Biophys. 2010, 493, 3–12. 10.1016/j.abb.2009.07.001. 19583966 PMC2812590

[ref57] Roston D.; Kohen A. A Critical Test of the “Tunneling and Coupled Motion” Concept in Enzymatic Alcohol Oxidation. J. Am. Chem. Soc. 2013, 135, 13624–13627. 10.1021/ja405917m. 24020836 PMC3818283

[ref58] Luo J.; Bruice T. C. Low-Frequency Normal Modes in Horse Liver Alcohol Dehydrogenase and Motions of Residues Involved in the Enzymatic Reaction. Biophys. Chem. 2007, 126, 80–85. 10.1016/j.bpc.2006.05.009. 16737770

[ref59] Samuel J.; Tanner M. E. Mechanistic Aspects of Enzymatic Carbohydrate Epimerization. Nat. Prod. Rep. 2002, 19, 261–277. 10.1039/b100492l. 12137277

[ref60] Thibodeaux C. J.; Melançon C. E.; Liu H. W. Unusual Sugar Biosynthesis and Natural Product Glycodiversification. Nature 2007, 446, 1008–1016. 10.1038/nature05814. 17460661

[ref61] Frey P. A.; Hegeman A. D. Chemical and Stereochemical Actions of UDP-Galactose 4-Epimerase. Acc. Chem. Res. 2013, 46, 1417–1426. 10.1021/ar300246k. 23339688

[ref62] Hallis T. M.; Zhao Z.; Liu H. W. New Insights into the Mechanism of CDP-D-Tyvelose 2-Epimerase: An Enzyme-Catalyzing Epimerization at an Unactivated Stereocenter. J. Am. Chem. Soc. 2000, 122, 10493–10503. 10.1021/ja0022021.

[ref63] Thoden J. B.; Henderson J. M.; Fridovich-Keil J. L.; Holden H. M. Structural Analysis of the Y299C Mutant of *Escherichia coli* UDP-Galactose 4-Epimerase. Teaching an Old Dog New Tricks. J. Biol. Chem. 2002, 277, 27528–27534. 10.1074/jbc.M204413200. 12019271

[ref64] Borg A.; Dennig A.; Weber H.; Nidetzky B. Mechanistic Characterization of UDP-Glucuronic Acid 4-Epimerase. FEBS J. 2021, 288, 1163–1178. 10.1111/febs.15478. 32645249 PMC7984243

[ref65] Allard S. T. M.; Giraud M. F.; Naismith J. H. Epimerases: Structure, Function and Mechanism. Cell. Mol. Life Sci. 2001, 58, 1650–1665. 10.1007/PL00000803. 11706991 PMC11337284

[ref66] Van Overtveldt S.; Verhaeghe T.; Joosten H. J.; van den Bergh T.; Beerens K.; Desmet T. A Structural Classification of Carbohydrate Epimerases: From Mechanistic Insights to Practical Applications. Biotechnol. Adv. 2015, 33, 1814–1828. 10.1016/j.biotechadv.2015.10.010. 26505535

[ref67] Tanner M. E. Understanding Nature’s Strategies for Enzyme-Catalyzed Racemization and Epimerization. Acc. Chem. Res. 2002, 35, 237–246. 10.1021/ar000056y. 11955052

[ref68] Thoden J. B.; Hegeman A. D.; Wesenberg G.; Chapeau M. C.; Frey P. A.; Holden H. M. Structural Analysis of UDP-Sugar Binding to UDP-Galactose 4-Epimerase from *Escherichia coli*. Biochemistry 1997, 36, 6294–6304. 10.1021/bi970025j. 9174344

[ref69] Borg A. J. E.; Beerens K.; Pfeiffer M.; Desmet T.; Nidetzky B. Stereo-Electronic Control of Reaction Selectivity in Short-Chain Dehydrogenases: Decarboxylation, Epimerization, and Dehydration. Curr. Opin. Chem. Biol. 2021, 61, 43–52. 10.1016/j.cbpa.2020.09.010. 33166830

[ref70] Tsai S. C.; Klinman J. P. Probes of Hydrogen Tunneling with Horse Liver Alcohol Dehydrogenase at Subzero Temperatures. Biochemistry 2001, 40, 2303–2311. 10.1021/bi002075l. 11329300

[ref71] Tuñón I.; Laage D.; Hynes J. T. Are There Dynamical Effects in Enzyme Catalysis? Some Thoughts Concerning the Enzymatic Chemical Step. Arch. Biochem. Biophys. 2015, 582, 42–55. 10.1016/j.abb.2015.06.004. 26087289 PMC4560206

[ref72] Rapp C.; Van Overtveldt S.; Beerens K.; Weber H.; Desmet T.; Nidetzky B. Expanding the Enzyme Repertoire for Sugar Nucleotide Epimerization: The CDP-Tyvelose 2-Epimerase from Thermodesulfatator Atlanticus for Glucose/Mannose Interconversion. Appl. Environ. Microbiol. 2021, 87, 1–14. 10.1128/AEM.02131-20. PMC7851689 33277270

[ref73] Van Overtveldt S.; Da Costa M.; Gevaert O.; Joosten H. J.; Beerens K.; Desmet T. Determinants of the Nucleotide Specificity in the Carbohydrate Epimerase Family 1. Biotechnol. J. 2020, 15, 2000132 10.1002/biot.202000132. 32761842

[ref74] Hallis T. M.; Liu H. W. Mechanistic Studies of the Biosynthesis of Tyvelose: Purification and Characterization of CDP-D-Tyvelose 2-Epimerase. J. Am. Chem. Soc. 1999, 121, 6765–6766. 10.1021/ja991465w.

[ref75] Kavanagh K. L.; Jörnvall H.; Persson B.; Oppermann U. Medium- and Short-Chain Dehydrogenase/Reductase Gene and Protein Families: The SDR Superfamily: Functional and Structural Diversity within a Family of Metabolic and Regulatory Enzymes. Cell. Mol. Life Sci. 2008, 65, 3895–3906. 10.1007/s00018-008-8588-y. 19011750 PMC2792337

[ref76] Jörnvall H.; Hedlund J.; Bergman T.; Oppermann U.; Persson B. Superfamilies SDR and MDR: From Early Ancestry to Present Forms. Emergence of Three Lines, a Zn-Metalloenzyme, and Distinct Variabilities. Biochem. Biophys. Res. Commun. 2010, 396, 125–130. 10.1016/j.bbrc.2010.03.094. 20494124

[ref77] Tanaka N.; Nonaka T.; Nakamura K.; Hara A. SDR Structure, Mechanism of Action, and Substrate Recognition. Curr. Org. Chem. 2001, 5, 89–111. 10.2174/1385272013375751.

[ref78] Nagel Z. D.; Meadows C. W.; Dong M.; Bahnson B. J.; Klinman J. Active Site Hydrophobic Residues Impact Hydrogen Tunneling Differently in a Thermophilic Alcohol Dehydrogenase at Optimal versus Nonoptimal Temperatures. Biochemistry 2012, 51, 4147–4156. 10.1021/bi3001352. 22568562 PMC3498984

[ref79] Maglia G.; Allemann R. K. Evidence for Environmentally Coupled Hydrogen Tunneling during Dihydrofolate Reductase Catalysis. J. Am. Chem. Soc. 2003, 125, 13372–13373. 10.1021/ja035692g. 14583029

[ref80] Pang J.; Pu J.; Gao J.; Truhlar D. G.; Allemann R. K. Hydride Transfer Reaction Catalyzed by Hyperthermophilic Dihydrofolate Reductase Is Dominated by Quantum Mechanical Tunneling and Is Promoted by Both Inter- and Intramonomeric Correlated Motions. J. Am. Chem. Soc. 2006, 128, 8015–8023. 10.1021/ja061585l. 16771517

[ref81] Loveridge E. J.; Hroch L.; Hughes R. L.; Williams T.; Davies R. L.; Angelastro A.; Luk L. Y. P.; Maglia G.; Allemann R. K. Reduction of Folate by Dihydrofolate Reductase from *Thermotoga maritima*. Biochemistry 2017, 56, 1879–1886. 10.1021/acs.biochem.6b01268. 28319664

[ref82] Luk L. Y. P.; Loveridge E. J.; Allemann R. K. Protein Motions and Dynamic Effects in Enzyme Catalysis. Phys. Chem. Chem. Phys. 2015, 17, 30817–30827. 10.1039/C5CP00794A. 25854702

[ref83] Liu Y.; Thoden J. B.; Kim J.; Berger E.; Gulick A. M.; Ruzicka F. J.; Holden H. M.; Frey P. A. Mechanistic Roles of Tyrosine 149 and Serine 124 in UDP-Galactose 4-Epimerase from *Escherichia coli*. Biochemistry 1997, 36, 10675–10684. 10.1021/bi970430a. 9271498

[ref84] Cook P. F.; Cleland W. W. Enzyme Kinetics and Mechanism; Taylor and Francis: New York, 2007.

[ref85] Klinman J. P.; Kohen A. Hydrogen Tunneling Links Protein Dynamics to Enzyme Catalysis. Annu. Rev. Biochem. 2013, 82, 471–496. 10.1146/annurev-biochem-051710-133623. 23746260 PMC4066974

[ref86] Karsten W. E.; Cook P. F.; Lai C. J. Inverse Solvent Isotope Effects in the NAD-Malic Enzyme Reaction Are the Result of the Viscosity Difference between D_2_O and H_2_O: Implications for Solvent Isotope Effect Studies. J. Am. Chem. Soc. 1995, 117, 5914–5918. 10.1021/ja00127a002.

[ref87] Ma G.; Dong L.; Liu Y. Insights into the Catalytic Mechanism of dTDP-Glucose 4,6-Dehydratase from Quantum Mechanics/Molecular Mechanics Simulations. RSC Adv. 2014, 4, 35449 10.1039/C4RA04406A.

[ref88] Nam Y. W.; Nishimoto M.; Arakawa T.; Kitaoka M.; Fushinobu S. Structural Basis for Broad Substrate Specificity of UDP-Glucose 4-Epimerase in the Human Milk Oligosaccharide Catabolic Pathway of *Bifidobacterium longum*. Sci. Rep. 2019, 9, 11081 10.1038/s41598-019-47591-w. 31366978 PMC6668579

[ref89] Major L. L.; Wolucka B. A.; Naismith J. H. Structure and Function of GDP-Mannose-3′,5′-Epimerase: An Enzyme Which Performs Three Chemical Reactions at the Same Active Site. J. Am. Chem. Soc. 2005, 127, 18309–18320. 10.1021/ja056490i. 16366586 PMC3315049

[ref90] Fushinobu S. Molecular Evolution and Functional Divergence of UDP-Hexose 4-Epimerases. Curr. Opin. Chem. Biol. 2021, 61, 53–62. 10.1016/j.cbpa.2020.09.007. 33171387

[ref91] Stawoska I.; Dudzik A.; Wasylewski M.; Jemioła-Rzemińska M.; Skoczowski A.; Strzałka K.; Szaleniec M. DFT-Based Prediction of Reactivity of Short-Chain Alcohol Dehydrogenase. J. Comput.-Aided Mol. Des. 2017, 31, 587–602. 10.1007/s10822-017-0026-5. 28550607 PMC5487757

[ref92] Wuxiuer Y.; Morgunova E.; Cols N.; Popov A.; Karshikoff A.; Sylte I.; Gonzàlez-Duarte R.; Ladenstein R.; Winberg J.-O. An Intact Eight-Membered Water Chain in Drosophilid Alcohol Dehydrogenases Is Essential for Optimal Enzyme Activity. FEBS J. 2012, 279, 2940–2956. 10.1111/j.1742-4658.2012.08675.x. 22741949

[ref93] Benach J.; Winberg J.-O.; Svendsen J.-S.; Atrian S.; Gonzàlez-Duarte R.; Ladenstein R. *Drosophila* Alcohol Dehydrogenase: Acetate–Enzyme Interactions and Novel Insights into the Effects of Electrostatics on Catalysis. J. Mol. Biol. 2005, 345, 579–598. 10.1016/j.jmb.2004.10.028. 15581900

[ref94] Plapp B. V.; Savarimuthu B. R.; Ferraro D. J.; Rubach J. K.; Brown E. N.; Ramaswamy S. Horse Liver Alcohol Dehydrogenase: Zinc Coordination and Catalysis. Biochemistry 2017, 56, 3632–3646. 10.1021/acs.biochem.7b00446. 28640600 PMC5518280

[ref95] Burke J. R.; Frey P. A. The Importance of Binding Energy in Catalysis of Hydride Transfer by UDP-Galactose 4-Epimerase: A Carbon-13 and Nitrogen-15 NMR and Kinetic Study. Biochemistry 1993, 32, 13220–13230. 10.1021/bi00211a034. 8241177

[ref96] Berger E.; Arabshahi A.; Wei Y.; Schilling J. F.; Frey P. A. Acid - Base Catalysis by UDP-Galactose 4-Epimerase: Correlations of Kinetically Measured Acid Dissociation Constants with Thermodynamic Values for Tyrosine 149. Biochemistry 2001, 40, 6699–6705. 10.1021/bi0104571. 11380265

[ref97] Swanson B. A.; Frey P. A. Identification of Lysine 153 as a Functionally Important Residue in UDP-Galactose 4-Epimerase from *Escherichia coli*. Biochemistry 1993, 32, 13231–13236. 10.1021/bi00211a035. 8241178

[ref98] Cleland W. W. Determination of Equilibrium Isotope Effects by the Equilibrium Perturbation Method. Methods Enzymol. 1982, 87, 641–646. 10.1016/S0076-6879(82)87034-1. 6757652

[ref99] Cook P. F.; Blanchard J. S.; Cleland W. W. Primary and Secondary Deuterium Isotope Effects on Equilibrium Constants for Enzyme-Catalyzed Reactions. Biochemistry 1980, 19, 4853–4858. 10.1021/bi00562a023. 7000186

[ref100] Cook P. F. Mechanism from Isotope Effects. Isot. Environ. Health Stud. 1998, 34, 3–17. 10.1080/10256019708036327. 9854842

[ref101] Quinn D.; Sutton L. Enzyme Mechanism from Isotope Effects; Cook P., Ed.; CRC Press, 1991.

[ref102] Fernandez P. L.; Murkin A. S. Inverse Solvent Isotope Effects in Enzyme-Catalyzed Reactions. Molecules 2020, 25, 1933 10.3390/molecules25081933. PMC7221790 32326332

[ref103] Quartararo C. E.; Blanchard J. S. Kinetic and Chemical Mechanism of Malate Synthase from *Mycobacterium tuberculosis*. Biochemistry 2011, 50, 6879–6887. 10.1021/bi2007299. 21728344 PMC3153559

[ref104] Cleland W. W. Low-Barrier Hydrogen Bonds and Enzymatic Catalysis. Arch. Biochem. Biophys. 2000, 382, 1–5. 10.1006/abbi.2000.2011. 11051090

[ref105] Sekhar V. C.; Plapp B. V. Rate Constants for a Mechanism Including Intermediates in the Interconversion of Ternary Complexes by Horse Liver Alcohol Dehydrogenase. Biochemistry 1990, 29, 4289–4295. 10.1021/bi00470a005. 2161681

[ref106] Fitzpatrick P. F. Combining solvent isotope effects with substrate isotope effects in mechanistic studies of alcohol and amine oxidation by enzymes. Biochim. Biophys. Acta 2015, 1854, 1746–1755. 10.1016/j.bbapap.2014.10.020. 25448013 PMC4416078

[ref107] Truhlar D. G.; Kohen A. Convex Arrhenius Plots and Their Interpretation. Proc. Natl. Acad. Sci. U. S. A. 2001, 98, 848–851. 10.1073/pnas.98.3.848. 11158559 PMC14672

[ref108] Glowacki D. R.; Harvey J. N.; Mulholland A. J. Taking Ockham’s Razor to Enzyme Dynamics and Catalysis. Nat. Chem. 2012, 4, 169–176. 10.1038/nchem.1244. 22354430

[ref109] Klinman J. P.; Offenbacher A. R. Understanding Biological Hydrogen Transfer Through the Lens of Temperature Dependent Kinetic Isotope Effects. Acc. Chem. Res. 2018, 51, 1966–1974. 10.1021/acs.accounts.8b00226. 30152685 PMC6258190

[ref110] Nagel Z. D.; Klinman J. P. A 21st Century Revisionist’s View at a Turning Point in Enzymology. Nat. Chem. Biol. 2009, 5, 543–550. 10.1038/nchembio.204. 19620995

[ref111] Marcus R. A.; Sutin N. Electron Transfers in Chemistry and Biology. Biochim. Biophys. Acta, Rev. Bioenergy 1985, 811, 265–322. 10.1016/0304-4173(85)90014-X.

[ref112] Masgrau L.; Roujeinikova A.; Johannissen L. O.; Hothi P.; Basran J.; Ranaghan K. E.; Mulholland A. J.; Sutcliffe M. J.; Scrutton N. S.; Leys D. Atomic Description of an Enzyme Reaction Dominated by Proton Tunneling. Science 2006, 312, 237–241. 10.1126/science.1126002. 16614214

[ref113] Lu Y.; Wilhelm S.; Bai M.; Maness P.; Ma L. Replication of the Enzymatic Temperature Dependency of the Primary Hydride Kinetic Isotope Effects in Solution: Caused by the Protein-Controlled Rigidity of the Donor–Acceptor Centers?. Biochemistry 2019, 58, 4035–4046. 10.1021/acs.biochem.9b00574. 31478638

[ref114] Islam Z.; Strutzenberg T. S.; Ghosh A. K.; Kohen A. Activation of Two Sequential H Transfers in the Thymidylate Synthase Catalyzed Reaction. ACS Catal. 2015, 5, 6061–6068. 10.1021/acscatal.5b01332. 26576323 PMC4643671

[ref115] Singh P.; Sen A.; Francis K.; Kohen A. Extension and Limits of the Network of Coupled Motions Correlated to Hydride Transfer in Dihydrofolate Reductase. J. Am. Chem. Soc. 2014, 136, 2575–2582. 10.1021/ja411998h. 24450297 PMC3985941

[ref116] Basran J.; Patel S.; Sutcliffe M. J.; Scrutton N. S. Importance of Barrier Shape in Enzyme-Catalyzed Reactions. J. Biol. Chem. 2001, 276, 6234–6242. 10.1074/jbc.M008141200. 11087744

[ref117] Behiry E. M.; Ruiz-Pernia J. J.; Luk L.; Tuñón I.; Moliner V.; Allemann R. K. Isotope Substitution of Promiscuous Alcohol Dehydrogenase Reveals the Origin of Substrate Preference in the Transition State. Angew. Chem., Int. Ed. 2018, 57, 3128–3131. 10.1002/anie.201712826. PMC5861672 29341402

[ref118] Cleland W. W. The Enzymes; Sigman D. S., Ed.; Academic Press: San Diego, 1990; Vol. 19.

