# Supplementary material for: Hydride Transfer Mechanism of Enzymatic Sugar Nucleotide C2 Epimerization Probed with a Loose-Fit CDP-Glucose Substrate
Source: ACS Catal. 2022 May 25;12(12):6816–30. doi: 10.1021/acscatal.2c00257 (PMC9207888; doi:10.1021/acscatal.2c00257)
Supplement: Supplementary file 1 — cs2c00257_si_001.pdf [file cs2c00257_si_001.pdf]

SUPPORTING INFORMATION

Hydride Transfer Mechanism of Enzymatic Sugar Nucleotide C2 Epimerization Probed with a Loose-Fit CDP-Glucose Substrate

*Christian Rapp[1] and Bernd Nidetzky[1,2]**

[1]Institute of Biotechnology and Biochemical Engineering, Graz University of Technology, Petersgasse 10-12/1, 8010 Graz, Austria.

[2]Austrian Centre of Industrial Biotechnology (acib), Petersgasse 14, 8010 Graz, Austria.

*Corresponding author: bernd.nidetzky@tugraz.at

Number of pages: 17

Number of tables: 2

Number of figures: 12

Number of schemes: 1

Table S1. KIE values for reaction of *Ta*CPa2E with saturating concentrations of CDP-Glc or CDP-[2-^{2}H]Glc in the temperature range of 293.15 - 353.15 K (20-80 °C). V_H: initial reaction rate of CDP-Glc (min^{-1}) and V_D: initial reaction rate of CDP-[2-^{2}H]Glc (min^{-1}). The pH was 7.5.

**T** (K)	**KIE** ($V_H V_D^{-1}$)
293.15	2.20 ± 0.75
298.15	3.20 ± 0.86
303.15	3.11 ± 0.46
308.15	3.80 ± 0.46
313.15	6.13 ± 0.19
318.15	5.51 ± 0.32
323.15	5.63 ± 0.27
333.15	4.29 ± 0.28
343.15	4.09 ± 0.25
353.15	3.34 ± 0.09

Table S2: DNA primer sequences used for construction of the *Ta*CPa2E_Y164F variant. The introduced mutations are underlined.

Name	Mutation	DNA primer (5' – 3')
*Ta*CPa2E-fwd	Y164F	TTTCATTCACCG<u>TTT</u>GGTTGTAGCAAAGGT
*Ta*CPa2E-rev	Y164F	ACCTTTGCTACAACC<u>AAA</u>CGGTGAATGAAA

Linear PCR amplification involving three cycles with forward and reverse primers applied separately was carried out at first with parameters set to: initial denaturation: 30 sec/98 °C; 3 cycles amplification: 10 sec/98 °C for denaturation, 15 sec/55 °C for primer annealing and 6 min/72 °C extension time with a final extension of 5 min/72 °C. Afterwards, PCR mixtures were combined mixing forward and reverse primer solutions and divided into two mixtures up to a final volume of 50 µl. The aforementioned PCR program was restarted for further 15 cycles. 10 U DpnI were added for residual template DNA removal and incubated for 16h at 37 °C followed by inactivation at 80°C for 20 min. Samples were centrifuged and PCR products analyzed by agarose gel electrophoresis. The PCR products were desalted and transformed into electro competent *E. coli* BL21 (DE3). Extracted plasmid DNA was sequenced with T7prom & T7term primers provided by LGC Genomics (Berlin, Germany). The correct construct was expressed as described elsewhere.[1]

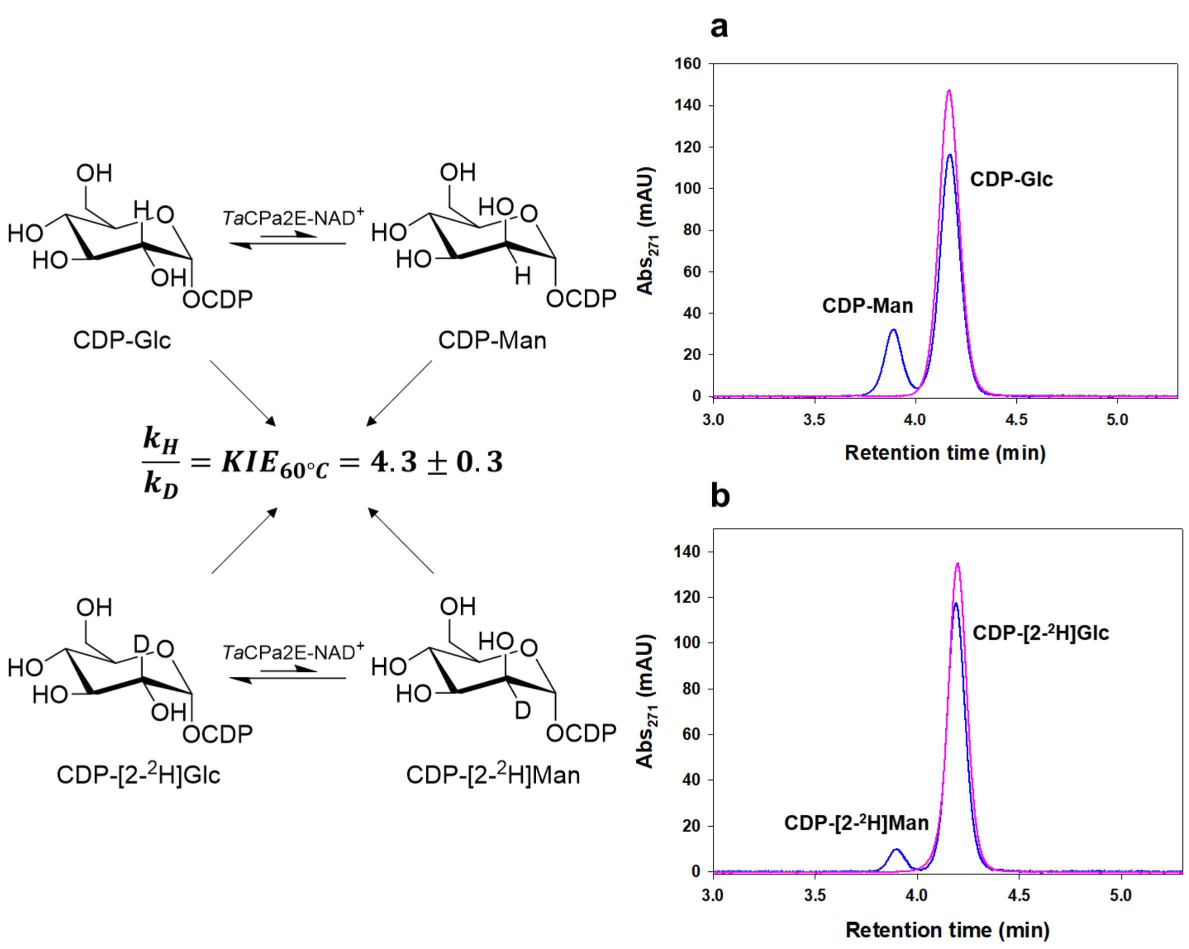

Figure S1. Determination of the noncompetitive KIE using CDP-[2-^{2}H]Glc or CDP-Glc. Depiction of reaction schemes with corresponding HPLC chromatograms of the reaction mixtures containing *Ta*CPa2E with (**a**) CDP-Glc or (**b**) CDP-[2-^{2}H]Glc. Reactions quenched after 15 min (blue) showed an additional peak assigned as CDP-Man or CDP-[2-^{2}H]Man. The control without enzyme is shown in pink. The KIE on k_{cat} shown was determined to 4.3 ± 0.3 (N = 4), where k_H: reaction rate of CDP -Glc (min^{-1}) and k_D: reaction rate of CDP-[2-^{2}H]Glc (min^{-1}). Reaction conditions: *Ta*CPa2E (1.0 mg mL^{-1}; 25.4 µM) at 60 °C in 100 mM MOPS buffer (pH 7.5) analyzed by HPLC (λ = 271 nm).

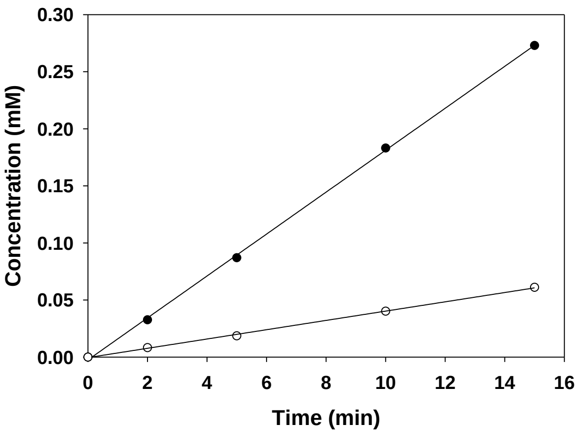

Figure S2. Exemplified comparison of the initial rates for the epimerization of CDP-[2-^{2}H]Glc (white circles) and CDP-Glc (black dots). A substrate concentration of 4.00 mM assured linear behavior over the relevant measurement time of 15 min. It was saturating at the steady state. Reactions shown were carried out in MOPS buffer (100 mM, pH 7.5) using *Ta*CPa2E (1.0 mg/mL; 25.4 µM) at 60°C.

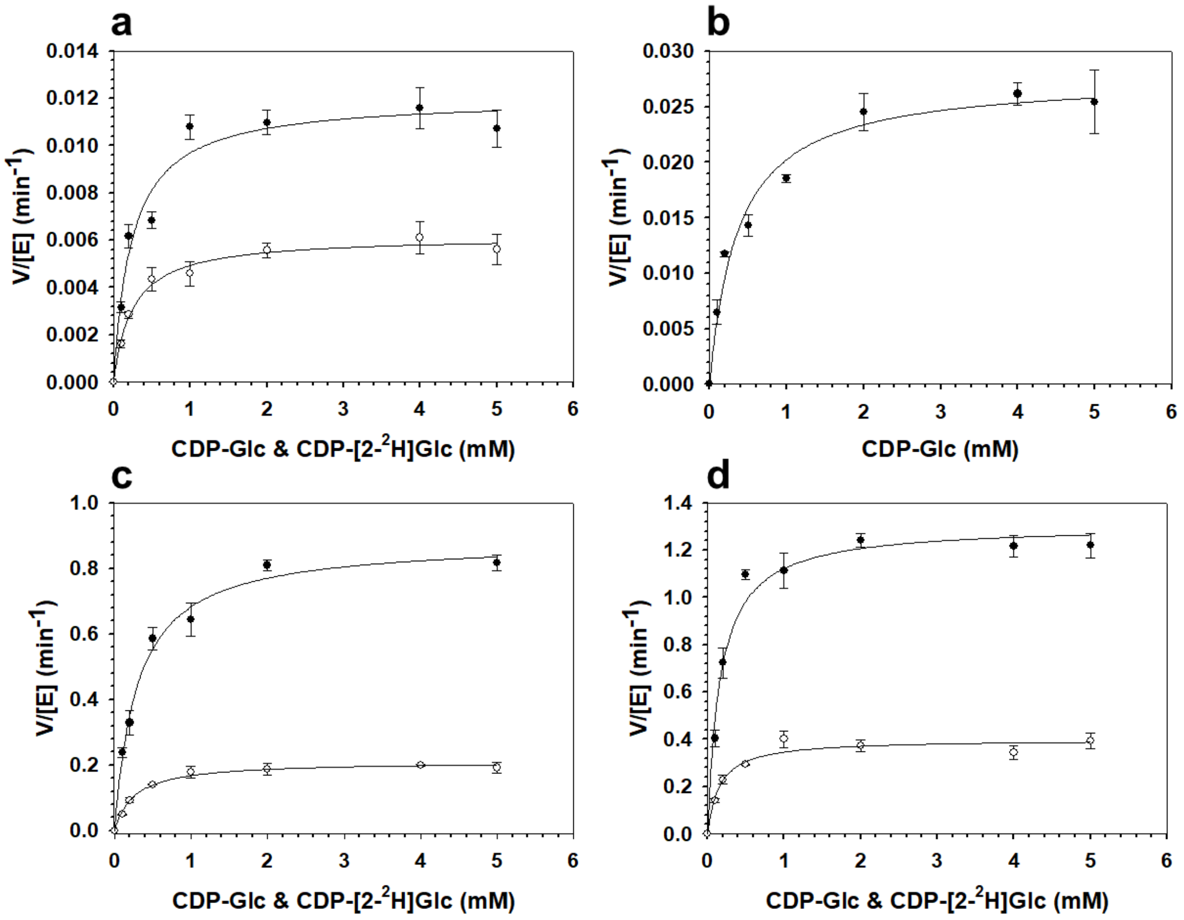

Figure S3. Kinetic data used for determination of $^{D}k_{cat}/K_M$ or $^{D2O}k_{cat}/K_M$ at various temperatures. Data obtained for CDP-[2-^{2}H]Glc (empty circle) and CDP-Glc (filled circle) was globally fit with the equation (Eq. 6) described in the Materials & Methods section. Kinetic parameters for the epimerization at (**a**) 20 °C in MOPS-^1H_2O, (**b**) 20 °C in MOPS-^2H_2O, (**c**) 60 °C in MOPS-^1H_2O and (**d**) 80 °C in MOPS-^1H_2O are presented in Tables 1 and 3. The pL was 7.5. The error bars show the S.D. from N ≥ 3 independent determinations.

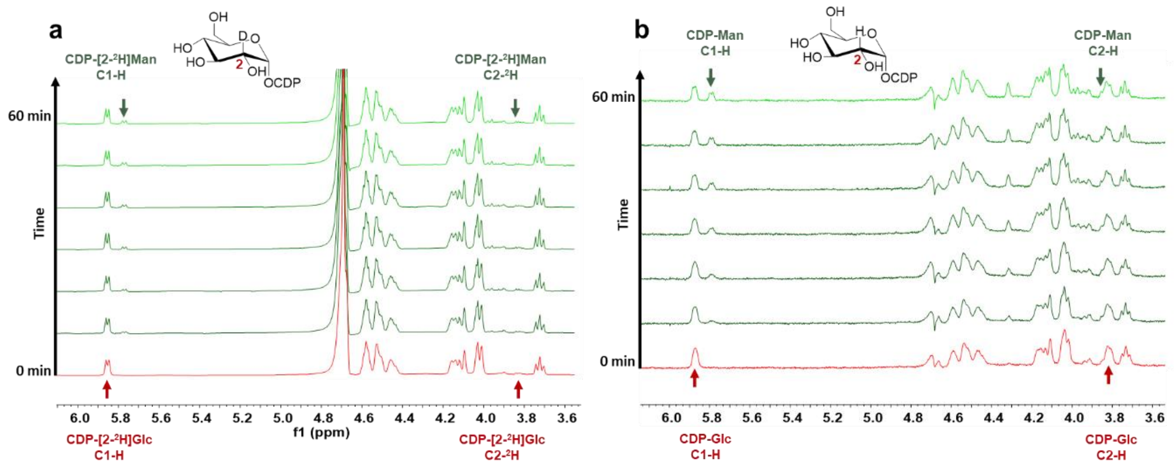

Figure S4. Complementing full spectrum of in-situ ^{1}H-NMR measurements applying *Ta*CPa2E and CDP-[2-^{2}H]Glc (**a**) or CDP-Glc (**b**). C2 signals and signals for the anomeric region are highlighted in red and green, respectively. For reaction conditions, see Figure 1 in the main text.

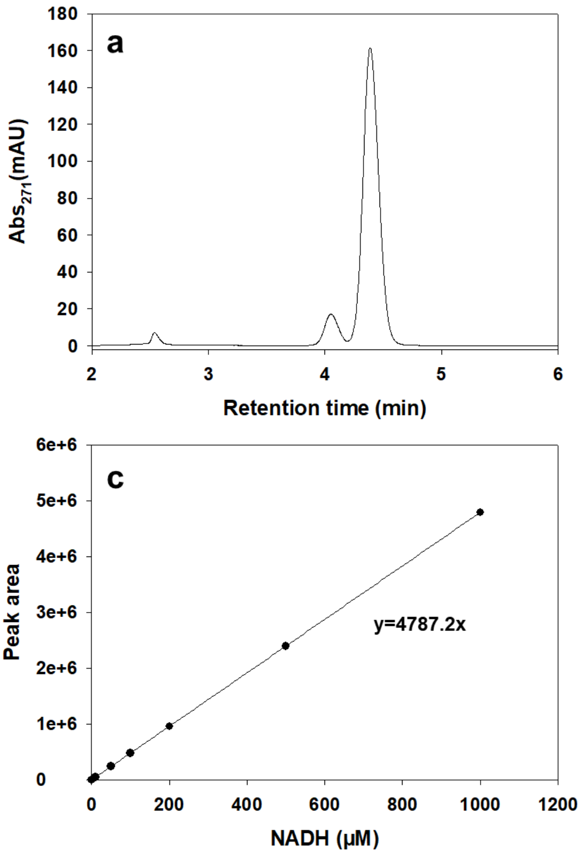

Figure S5. NADH quantification and HPLC analysis of flow-through and supernatant to assure an unchanged reaction equilibrium of CDP-Glc/CDP-Man in the rapid-quench assay. **a.** Reaction mixture after 15 min when quenched with methanol and enzyme removed. **b.** Flow-through of the reaction mixture after cooling and acidification. Conversions (%) to CDP-Man were identical in the flow-through and supernatant. Retention times: CDP-Man (4.1 min) and CDP-Glc (4.4 min). **c.** The extent of released NADH was determined based on the calibration curve shown. Defined NADH standard solutions (10-1000 µM) were prepared in double-distilled water and directly used for HPLC analysis at a UV-absorbance of 271 nm.

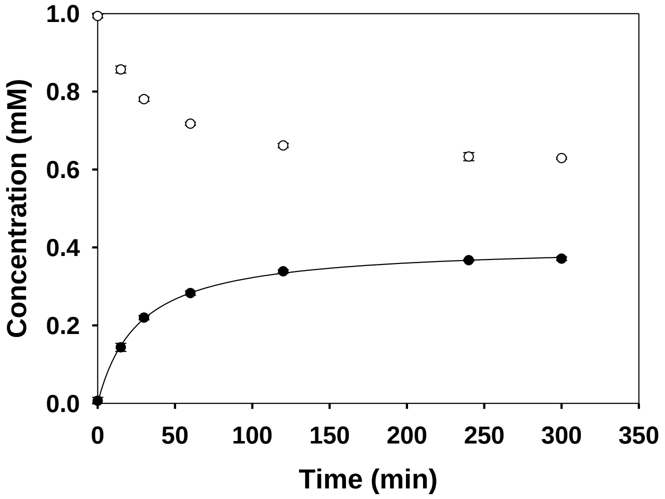

Figure S6. Determination of the reaction equilibrium for *Ta*CPa2E catalyzed conversion of CDP-[2-^{2}H]Glc. Time course for the conversion of CDP-[2-^{2}H]Glc (1.00 mM; empty circles) to CDP-[2-^{2}H]Man (filled circles) using *Ta*CPa2E (2.0 mg mL^{-1}; 50.8 µM) at 60 °C in 100 mM MOPS buffer (pH 7.5) analyzed by HPLC (λ = 271 nm). The data set was fit to a two-parameter hyperbola f = ax/(b+x), in which a = 0.4010 was used to calculate K_{eq} = a/(1-a).

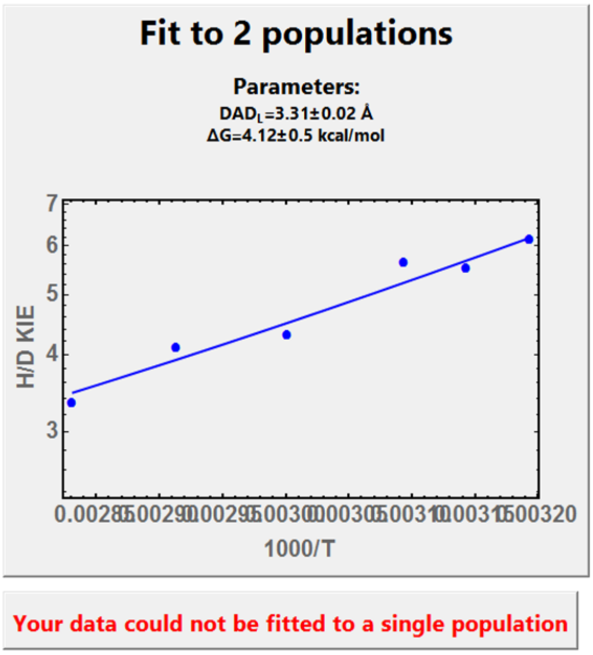

Figure S7. Screenshot of the DAD calculation for *Ta*CPa2E obtained by fitting hydrogen/deuterium (H/D) KIEs against the inverse temperature. The fit for *Ta*CPa2E in the temperature range of 40-80 °C exclusively resulted in a two-population distribution. DAD calculation was performed applying the program provided at: http://chemmath.chem.uiowa.edu/webMathematica/kohen/marcuslikemodel.html.

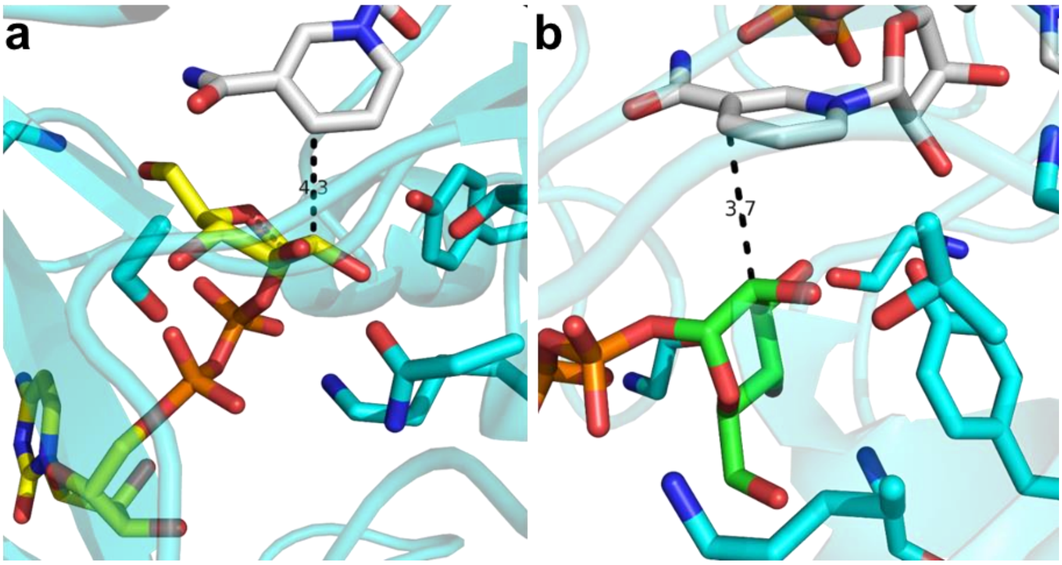

Figure S8. Donor-acceptor distance in the structure model of *Ta*CPa2E. CDP-Glc (**a**) and CDP-Man (**b**) obtained by ligand docking are shown. The DAD is considered to be the distance from C2 of the sugar moiety (H^- donor) to C4 of NAD^+ (H^- acceptor). DADs are indicated with dashed lines suggesting 4.3 Å for CDP-Glc and 3.7 Å for CDP-Man, respectively.

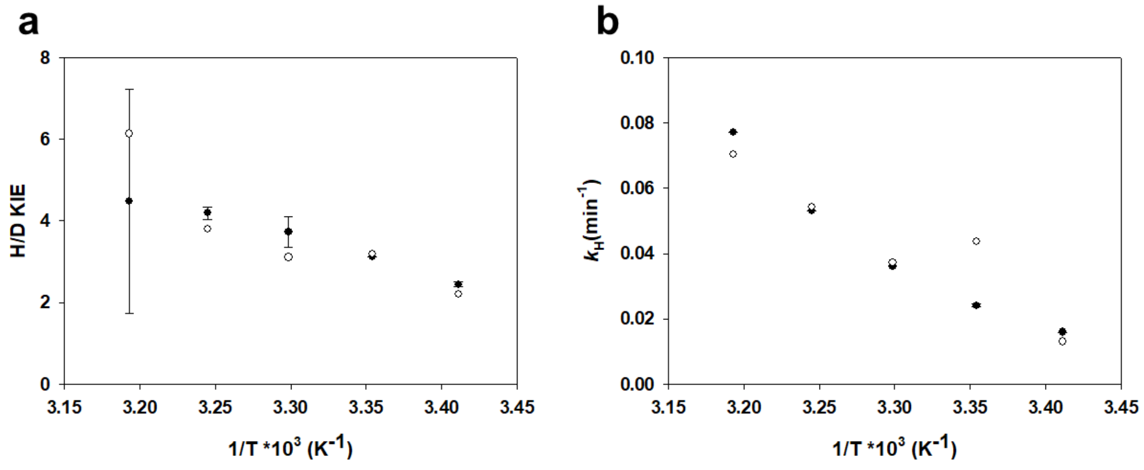

c

	VALUE	RANGE
$A_{H,\ 45\text{-}70°C}$	1736087751	constant
$A_{D,\ 45\text{-}70°C}$	37548527500	constant
$E_{A,\ H,\ 45\text{-}70°C}$	60214.98111	constant
$E_{A,\ D,\ 45\text{-}70°C}$	72937.76757	constant
**K**	1.034034707	variable
$A_{H,\ 20\text{-}40°C}$	0.018960932	variable; ≥0
$A_{D,\ 20\text{-}40°C}$	0.009143	variable; ≥0
$E_{A,\ 20\text{-}40°C}$	0	variable; ≥0
$(A_H/A_D)_{20\text{-}40°C}$	2.073893218	variable; ≤2.5

Figure S9. Two-state equilibrium model output with fitting parameters and set constraint values. Model (M; black dots) and experimental values (E; white circles) are overlaid and squared errors ($(M - E)^2$) shown as bars. Predictions obtained for the KIE (**a**) and k_H (**b**) are displayed. (**c**) Table showing parameters set or obtained after data fitting. Equations (7a) and (7b) of the main text were used. Error bars show the S.D. from N ≥ 4 independent determinations.

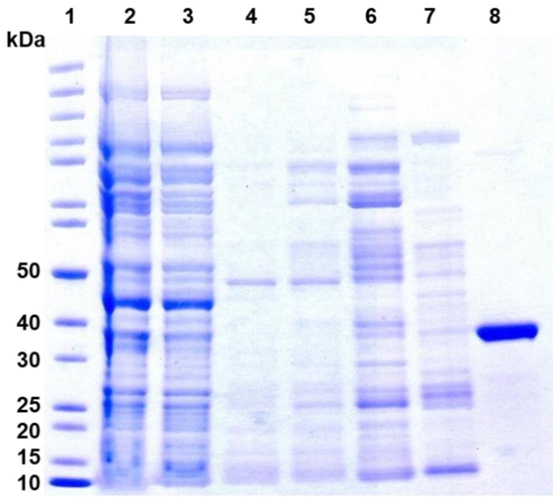

Figure S10. SDS polyacrylamide gel showing the purified *Ta*CPa2E_Y164F variant. Lane 1: molecular mass ladder, lanes 2-3: cell free extract, lanes 4-7: His-tag affinity chromatography flowthrough fractions, 8: concentrated *Ta*CPa2E_Y164F (MW = 39346 g mol^{-1}).

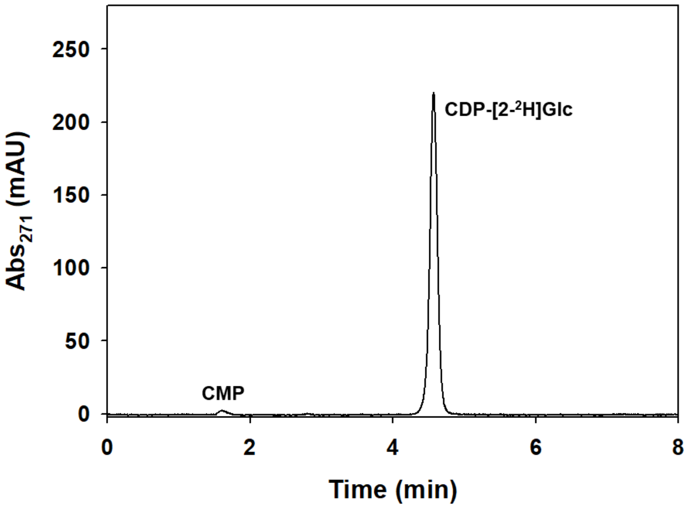

Figure S11. HPLC chromatogram of the purified CDP-[2-^{2}H]Glc.

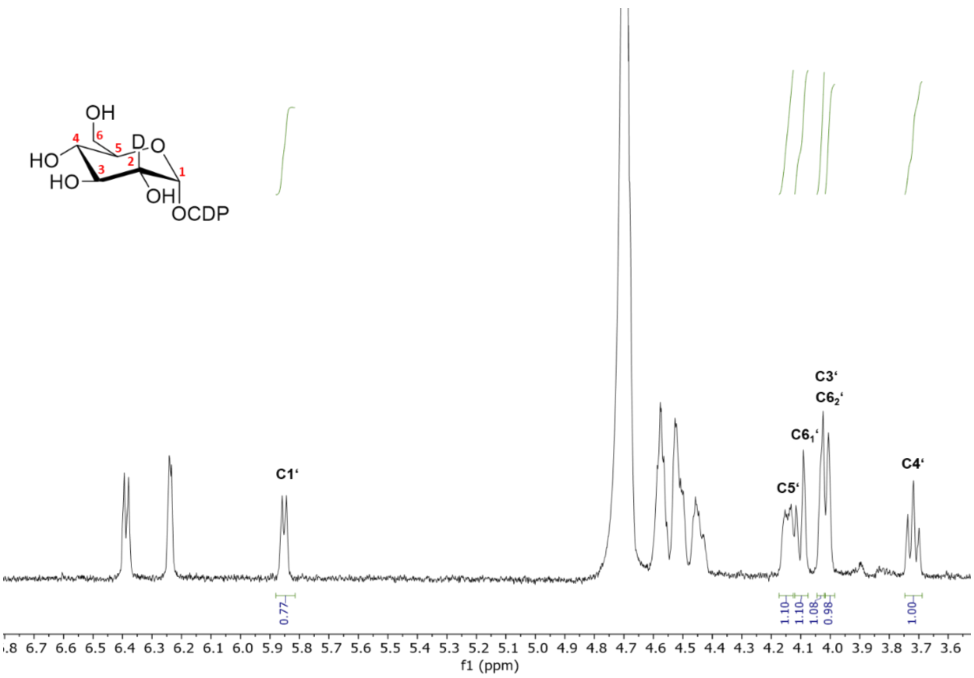

Figure S12. ^{1}H-NMR spectrum (500 MHz, D_2O) of CDP-[2-^{2}H]Glc in 50 mM potassium phosphate buffer (p^{2}H 7.5). δ 5.85 ppm (d, 1H), 4.15 ppm (m, 1H), 4.10 ppm (t, 1H), 4.00 ppm (d, 1H), 4.05 ppm (d, 1H), 3.72 ppm (t, 1H).

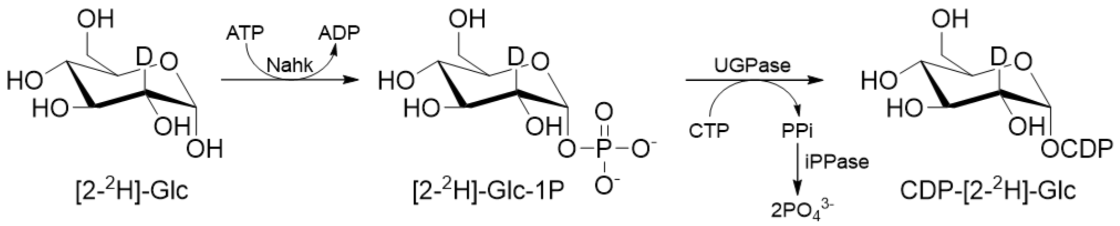

Scheme S1. Enzymatic synthesis of CDP-[2-^{2}H]Glc starting from [2-^{2}H]-Glc. N-acetylhexosamine 1- kinase (NahK) and ATP were used for anomeric phosphorylation of [2-^{2}H]-Glc. [2-^{2}H]-glucose-1-phosphate ([2-^{2}H]-Glc-1P) was incubated with CTP, UDP-glucose pyrophosphorylase (UGPase) and inorganic pyrophosphatase (IPPase) for the production of CDP-[2-^{2}H]Glc.

Reference

(1) Rapp, C.; Overtveldt, S. Van; Beerens, K.; Desmet, T.; Nidetzky, B. Expanding the Enzyme Repertoire for Sugar Nucleotide Epimerization: The CDP-Tyvelose 2-Epimerase from *Thermodesulfatator Atlanticus* for Glucose/Mannose Interconversion. *Appl. Environ. Microbiol.* **2021**, *87*, 1–14.
